# A Waterfowl Reproduction Model to Predict Carryover Effects of Oil Spills

**DOI:** 10.1002/ece3.74537

**Published:** 2026-11-04

**Authors:** Benjamin M. West, Mark L. Wildhaber, Wayne E. Thogmartin, Michael J. Hooper

**Affiliations:** ^1^ U.S. Geological Survey Columbia Environmental Research Center Columbia Missouri USA; ^2^ U.S. Geological Survey Upper Midwest Environmental Sciences Center La Crosse Wisconsin USA; ^3^ Department of Environmental Toxicology Texas Tech University Lubbock Texas USA

**Keywords:** carryover effects, mallard, migratory species, oil spill, reproduction, simulation modeling

## Abstract

For wintering and migrating birds, contamination from oil and other substances may delay arrival on the breeding grounds and lead to lower body mass upon arrival. These delays and losses of body mass could lead to smaller clutch sizes and lower propensity to renest. To determine potential effects of these consequences on breeding, we used an augmented version of an existing mallard ( *Anas platyrhynchos* ) reproduction model. By combining that reproduction model with the outputs of a previously developed migration and energetics model, we assessed carryover effects of simulated wintering ground oil spills on duckling production and other breeding season‐related population parameters. For simulated oil spills that occurred in January, reduced adult population and sublethal effects of migration delays both contributed to decreases in duckling production. For a simulated oil spill that occurred in March, sublethal effects from reduced body mass were the primary driver of decreased duckling production. By combining migration and energetics modeling with a reproduction model, this framework could provide the basis for a tool to generate quantitative estimates of lost reproduction resulting from carryover effects. Furthermore, this modeling framework could also be adapted for other avian species, facilitating assessments of reproductive loss in the context of Natural Resource Damage Assessments following contamination events.

## Graphical abstract

For wintering and migrating birds, contamination from oil and other substances may delay arrival on the breeding grounds and lead to lower body mass upon arrival. These delays and losses of body mass could lead to smaller clutch sizes and lower propensity to renest. By combining a mallard ( *Anas platyrhynchos* ) reproduction model with the outputs of a previously developed migration and energetics model, we assessed carryover effects of simulated wintering ground oil spills on duckling production and other breeding season‐related population parameters.

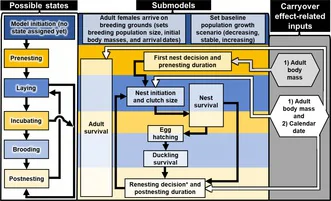

## 1 Introduction

Reproduction of birds can be negatively affected by environmental contamination from a variety of sources, including petroleum (King et al. 2021), mercury (Ackerman et al. 2016), pesticides (Fry 1995; Trijau et al. 2023; Martin et al. 2024), and industrial chemicals (Fry 1995). Contamination events or other disturbances may occur at any time during their annual cycle, leading to the potential for negative carryover effects for birds migrating from impacted sites to their breeding sites (Henkel et al. 2012; Legagneux et al. 2012; Montevecchi et al. 2012; Sedinger and Alisauskas 2014). Direct toxic effects are one source of decreased bird reproduction after contamination events (Fry 1995; Ackerman et al. 2016; King et al. 2021; Martin et al. 2024), but other consequences of contamination events may also negatively affect reproductive outputs.

In particular, oil spills may negatively impact bird reproduction in multiple ways. If the spill occurs near the breeding grounds, oil can be absorbed through eggshells and cause direct mortality of the eggs (King et al. 2021). Oil spills are also known to cause negative energetic effects on adult birds, which may lead to birds arriving at the breeding grounds with delayed arrival dates and/or lower body mass (Henkel et al. 2012; West et al. 2026a). In multiple bird species, individuals arriving late to the breeding grounds and/or in poor condition tend to have lower reproductive output or may forgo breeding (Rowe et al. 1994; Chastel et al. 1995; Devries et al. 2008; Milenkaya et al. 2015; Sedinger and Alisauskas 2014). There are multiple ways in which oiling can upset the energetic balance of birds and cause mass loss, including increasing thermoregulatory costs (Mathewson et al. 2018; West et al. 2022), increasing the cost of flight (Maggini et al. 2017; Perez et al. 2017), decreasing foraging rate (Burger and Tsipoura 1998), or disrupting the ability of birds to accumulate fat stores through toxic effects (Bianchini and Morrissey 2018b). Recent simulation models examining the energetic effects on migratory birds show that the combination of those energetic effects is likely to lead to reduced body mass upon arrival at the breeding grounds (West et al. 2026a). Combined, these observations provide a linkage between oiling‐induced energetic and migration effects and negative effects on reproductive success. Energetics‐mediated migration delays after oil spills have been theorized (Henkel et al. 2012; West et al. 2022). However, to the best of our knowledge, there is so far only one observational study providing potential empirical evidence of oil‐related migration delays at the time of writing (Bianchini and Morrissey 2018a).

Contaminant‐induced decrements in reproductive function serve as a direct measure of resource injury in regulatory actions taken in response to contaminant releases (Baker et al. 2020). Documenting the occurrence and extent of such injuries provides foundational data for the determination of damages attainable for restoration actions that compensate for losses of injured resources. In the 2010 *Deepwater Horizon* oil spill assessment, lost reproduction of birds was quantified as offspring that would have been produced by birds that died in the spill (Deepwater Horizon NRDA Trustees 2016). A similar adult mortality‐based approach to foregone reproduction had also been used for prior spills (Sperduto et al. 2003; Bird and Wildlife Technical Working Group 2007; USFWS et al. 2012). A variety of models were used to estimate avian mortality after *Deepwater Horizon*, but sublethal effects on reproduction were not quantified as part of the Natural Resource Damage Assessment (NRDA; Deepwater Horizon NRDA Trustees 2016). Because of the potential for lost reproduction due to contaminant‐induced effects on energetic balance and migration delays, accounting only for adult mortality could underestimate total reproductive loss resulting from oil spills. Additional modeling could be used to quantify those sublethal effect‐induced injuries in future NRDAs or similar contexts.

Due to its importance as a game species, the mallard ( *Anas platyrhynchos* ) has had a large amount of research invested in understanding factors influencing its reproduction (e.g., Krapu 1981; Cowardin et al. 1985; Hoekman et al. 2002; Devries et al. 2008). At least one existing mallard reproduction computer model, developed by Johnson et al. (1987), directly incorporates the effects of arrival date and adult female body mass on mallard reproduction. Mallards and other ducks, such as the common eider ( *Somateria mollissima* ), have also been the subject of studies that quantify the energetic effects of oiling, either through laboratory experiments (Munro Jenssen and Ekker 2016; Hartung 1967; McEwan and Koelink 1973) or computer modeling (West et al. 2022, 2026a; Tuarze et al. 2021). Mallards can also be directly affected by oil spills, though they do not tend to be the primary species affected by oceanic and coastal oil spills such as *Deepwater Horizon* (Deepwater Horizon NRDA Trustees 2016). The Deepwater Horizon NRDA Trustees (2016) estimated between 176 and 270 mallards were killed in that oil spill. Mallards were used as a surrogate species for all duck species in the NRDA for the 2004 *M/T Athos I*; the Bird and Wildlife Technical Working Group (2007) estimated direct mortality of 1574 adult ducks and lost production of 1350 additional individuals across all duck species. This combination of pre‐existing knowledge and computer models make mallards a strong candidate species to develop a framework for understanding how oil spill effects, such as mortality, migration delays, and reduced body condition, may affect migratory bird populations.

The proximate goal of this study was to combine the results of a migration and energetics model incorporating oil spills (West et al. 2026a) and an updated version of a stochastic mallard reproduction model (Johnson et al. 1987) to predict the effects of oil spills on a simulated mallard population. We specifically emphasized wintering ground oil spills and effects that carry over to the breeding season. We estimated and compared parameters that incorporated both lethal and sublethal effects of oiling, specifically estimated population growth rate and population‐level duckling production, that is, the number of ducklings that survived until fledging. We also attempted to isolate sublethal effects of oiling on reproductive output by assessing per‐adult duckling production rate, mean number of nesting attempts per adult female, and mean clutch size across all nests created by a population of adult female mallards. The ultimate goal of this effort was to demonstrate a modeling framework that could be used to assess both lethal consequences and sublethal carryover effects from oil spills on migratory birds.

## 2 Methods

We evaluated carryover effects of winter oil exposure on mallard reproduction by coupling two models: a mallard migration and energetics model (MMEM) and a reproduction model. The MMEM simulates wintering and spring migration, producing per‐bird arrival timing and body condition (lipid reserves and mass) at the breeding grounds (West et al. 2026a). These outputs initialized the reproduction model, which then simulated nesting behavior, survival of adult females, nests, and broods, and duckling production (see West et al. 2025 for model code). We assessed effects by comparing oiled simulations against matched unoiled baselines across a hierarchy of spill timings, oiling levels, and production scenarios.

### 2.1 Base Reproduction Model

To implement this framework, we began with a previously published reproduction model developed by Johnson et al. (1987). This simulation model is stochastic and process‐based. It tracks individual breeding female mallards throughout the breeding season as they produce zero, one, or more nests, attempting to survive and hatch one brood of ducklings for the season. Major model events include female mallards arriving at the breeding grounds, daily survival of breeding females, nest initiations, nest survival, and duckling survival. The Johnson et al. (1987) model also incorporates effects of date and body mass on reproduction, especially clutch size and renesting propensity. Inclusion of these effects was important to our selection of this model because oil spills may lead to lower body mass and/or delayed arrival dates for breeding birds (Henkel et al. 2012; West et al. 2022, 2026a).

To incorporate MMEM outputs and align the model with oil spill scenarios, we modified and augmented the Johnson et al. (1987) model in several ways. Instead of sampling initial adult female body masses and breeding ground arrival dates within the reproduction model, we used the masses and dates from the outputs of a mallard migration and energetics model (abbreviated as “MMEM,” West et al. 2026a, 2026b), reviewed in Section 2.2, “Migration and energetics model (MMEM).” For consistency with the MMEM, we only include adult (i.e., after second‐year) female mallards in our modeling efforts, whereas Johnson et al. (1987) modeled both yearling and adult females. In addition to updating reproduction processes, we modeled adult survival based on a study modeling how various factors affect female mallard breeding season survival in the Prairie Pothole Region (Arnold et al. 2012). One advantage of using the models of Arnold et al. (2012) is that they quantify the effects of body mass and date on adult survival, effects not included in the original Johnson et al. (1987) model. Another key feature of the Arnold et al. (2012) models is that they separate a mallard's reproductive season into discrete “reproductive periods” that we refer to simply as “states.” These states can be one of prenesting, laying, incubating, brooding, or postnesting (Figure 1); this last state refers to adults who have initiated at least one nest but were not actively tending a nest or brood due to prior reproductive failure. Additional changes made to the Johnson et al. (1987) model are detailed in the “Submodels” section within Appendix A and summarized in Appendix B.

**FIGURE 1:**
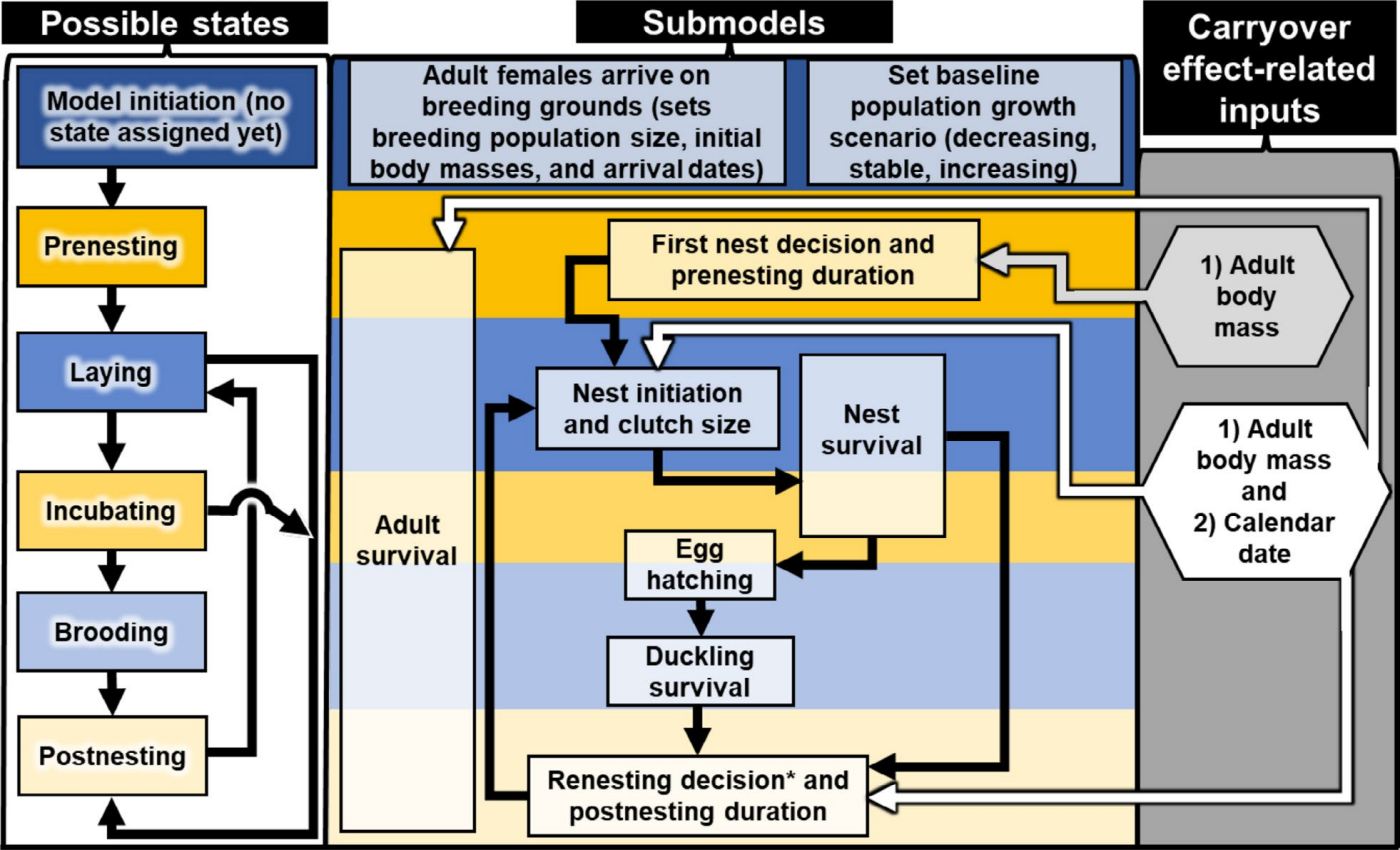
A simplified conceptual diagram describing a mallard reproduction model. “Possible states” apply to adult female mallards. In the “Submodels” section, the vertical position and background color correspond to states where a submodel is applied. Black arrows denote possible transitions between states or submodels. * = mallards that hatch a nest do not renest in this model.

In each simulation of the reproduction model, a replicate comprised a single breeding season with an initial population of up to 1000 adult female mallards capable of generating offspring as the model progressed. We conducted a total of 384,000 simulations; a breakdown of how we derived that number simulations is provided in the “Reproduction model scenarios and runs” section and visually represented in Figure 2. At the end of each simulation, we summarized the simulation across all adults and their offspring. The recorded results included overall survival rates for adults, nests, and ducklings and the number of nests attempted across all adults; for full model outputs, see West et al. (2025).

**FIGURE 2:**
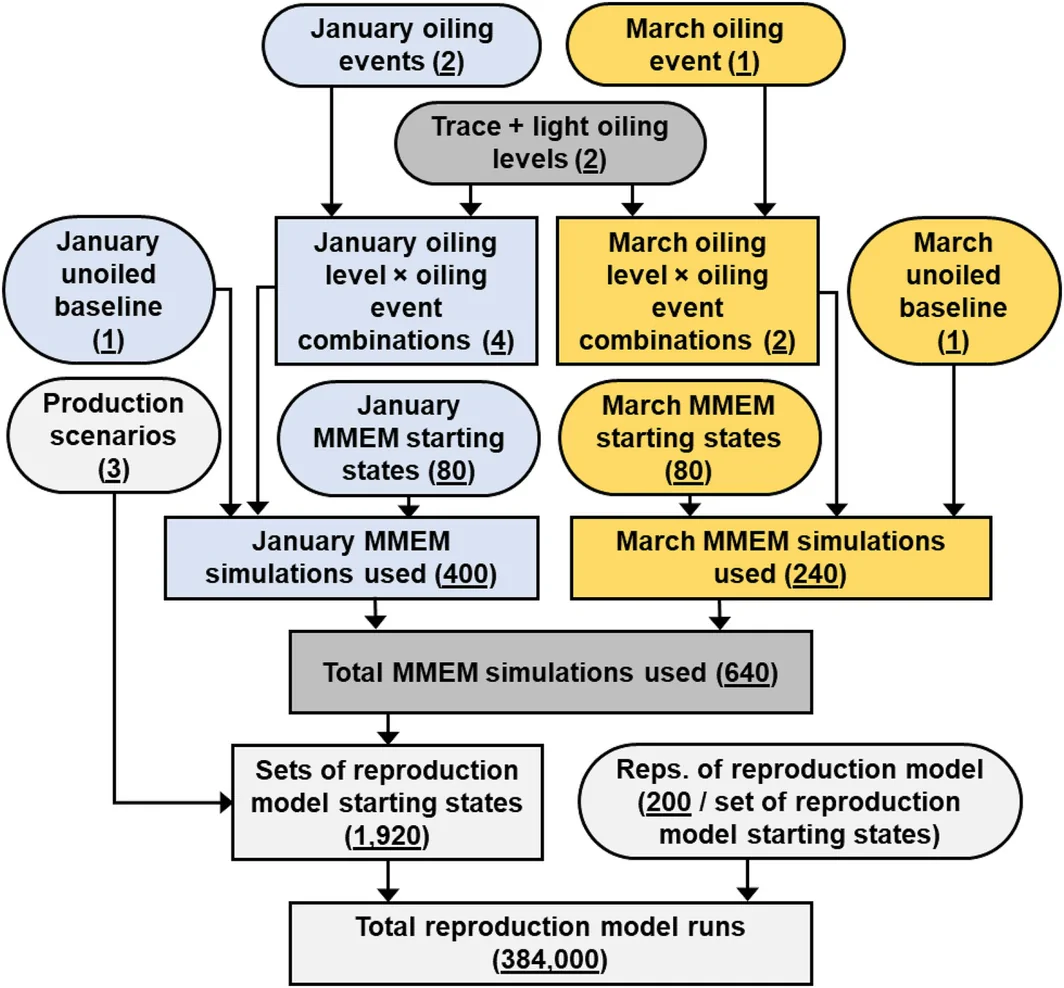
A flow chart showing the hierarchical relationships between and within a mallard (*Anas platyrhynchos*) reproduction model and a mallard migration and energetics model (MMEM) incorporating oil spill effects. Rounded shapes are basal factors within a model; rectangles are combinations of two or more basal factors. Underlined numbers in parentheses are the number of basal factors or combinations of basal factors. For fill colors, blue = related to a January oil spill in the MMEM, yellow = related to a March oil spill in the MMEM, darker gray = related to all oil spills in the MMEM, lighter gray = related to the mallard reproduction model. “Reps.” = replicates.

### 2.2 Mallard Migration and Energetics Model (MMEM)

#### 2.2.1 Overview

The primary goal of our mallard reproduction modeling effort was to estimate how timing of oil spills on the wintering grounds and level of feather oiling following those spills would affect reproductive output in mallards in a single breeding season immediately following a spill. To help accomplish this goal, we initialized the reproduction model using output data from a previously developed MMEM incorporating oil spills (West et al. 2026a, 2026b). Because MMEM outputs drive the reproduction model, we give a broad overview of MMEM components relevant to breeding outcomes below. We provide a more detailed summary in Appendix C, and full details can be found in West et al. (2026a), especially the detailed model summary in that manuscript's appendix.

The MMEM is implemented in three stages, each of which has multiple sources of stochasticity; see the appendix of West et al. (2026a) for complete detailing of which elements are stochastic within each stage. Stage 1 of the MMEM randomly generates spring migration paths and timings for mallard migrating from wintering area in northeastern Arkansas, USA to breeding areas in the Prairie Pothole Region. Stage 2 determines daily baseline (i.e., unoiled) energetic balances and body masses for adult female mallards following the migration paths and timings generated by the first stage. Stage 3 of the MMEM modifies them to create new simulations that incorporate the energetic and survival effects of oil spills on wintering and spring‐migrating adult female mallards (West et al. 2026a).

Across all stages, simulations start at a single wintering area in northeastern Arkansas, USA (West et al. 2026a). All simulations start on 23 January, and a simulation of 1000 adult female (i.e., after second year) mallards comprises one model unit. Mallards attempt to survive the remainder of winter and spring migration and migrate to a breeding area in the Prairie Pothole Region, accumulating energetic stores, specifically lipid reserves, needed for survival, migration, and reproduction. Birds have the potential to make one or more stopovers before arriving at their breeding area. The MMEM no longer tracks mallards after they arrive at their breeding area; the current reproduction modeling effort was made to fill that gap.

#### 2.2.2 Oiling Implementation

In addition to an unoiled baseline, mallards in the MMEM can experience one of two oiling levels during a designated period of active oiling: trace (≤ 5% of feather area oiled) or light (6% to 20% of feather area oiled). Those oiling levels are based on the categories used by the *M/T Athos I* and *Deepwater Horizon* oil spill assessments (Bird and Wildlife Technical Working Group 2007; USFWS 2015). Moderate (21% to 40% feather area oiled) and heavy (> 40% feather area oiled) oiling categories are not included in the West et al. (2026a) models because field observations show that birds experiencing those oiling levels seldom survive (Fallon et al. 2018; Paruk et al. 2019), and modeling results suggest that moderately oiled surviving birds likely experience substantial disruptions to their annual cycle (West et al. 2022). For simulations incorporating oil spill effects, individuals used in the reproduction model experienced energetic effects of oiling within the MMEM for all of thermoregulation, energetic cost of flight, and ability to gain energy from food.

The MMEM simulates three oiling events spanning two hypothetical oil spill timings. One spill timing starts on 23 January and the other starts on 6 March. Two oiling events correspond to the January spill timing; one does not alter migration timing, and the other has all mallards experience a 14‐day delay departing the wintering grounds. The March spill timing only had one oiling event that had no migration delay. Only one oiling event is applied to a given simulation. The first January oiling event is designed to test oiling effects under thermoregulatory stress; the second January oiling event is meant to test the combined effects of thermoregulatory stress and delayed arrival to the breeding grounds; and the March oiling event is meant to test effects of an oil spill on the wintering ground occurring temporally closer to the breeding season (West et al. 2026a). Migration delays are not included for all oiling events because migration delays from oiling are largely theoretical (Henkel et al. 2012; West et al. 2022); there are few studies that empirically assess or provide evidence for oiling affecting migration timing (cf. Bianchini and Morrissey 2018a).

For each oil spill timing, January or March, the MMEM has 80 replicate starting states, totaling 160 total starting states. Each state contains 1000 adult female mallards and includes initial body mass, initial lipid mass, and a migration schedule for each individual (West et al. 2026a, 2026b). Within a given spill timing, those replicates are used across all oiling levels and events, creating a repeated measures design that improves comparability among oiling levels. Full simulation hierarchy structure is detailed in Appendix C and visually represented in Figure 2.

### 2.3 Reproduction Model Scenarios and Runs

Using 640 unique MMEM simulation outputs as inputs for the reproduction model allowed us to meet our primary goal of estimating how the timing of oil spills and the intensity of oiling affect reproductive output in mallards. However, only conducting one reproduction model run per MMEM simulation created at least two issues, requiring us to conduct multiple reproduction model simulations per MMEM output.

The first issue occurred because mallard population growth rates are highly variable. Midcontinent North American (hereafter “Midcontinent”) mallards have experienced prominent periods of population declines from 1958 to 1965, 1970 to 1985, 1999 to 2005, and 2016 to ~2025 (USFWS 2025). Conversely, there have been prominent periods of Midcontinent mallard population increase from 1965 to 1970, 1992 to 1999, and 2005 to 2016 (USFWS 2025). These observations mean that Midcontinent mallards have variable population growth rates across decades. However, the default parameterization of Johnson et al. (1987) mallard reproduction model, along with other North American mallard population models and production studies (e.g., Amundson et al. 2013; Cowardin et al. 1985; Hoekman et al. 2002), generally represent declining mallard populations. Zafonte and Hampton (2005) show that magnitude of impacts on avian reproduction following events like oil spills can depend upon population‐level reproductive rate. Therefore, if reproduction‐driven variability exists in population growth rates, modeling that variability could be important in the context of assessing oil spill effects on avian populations.

To incorporate variability in population growth conditions, Zafonte and Hampton (2005) model low, medium, and high fecundity scenarios in their generalized avian population model. We adopted this three‐category approach, but we use the term “production scenarios” to be inclusive of breeding season mortality of adults and offspring. Note that our low, medium, and high categorizations were relative to the other production scenarios within our modeling framework. Though we used real‐world data to inform our production scenarios (see Appendix D), we did not necessarily assume that the modeled production scenarios had “low” or “high” duckling production compared to real‐life mallard populations. Our low production scenario did not alter the base reproduction model. The methods for how we derived parameterizations for medium and high production scenarios are contained in Appendix D. In general, we altered all of (1) adult daily survival rate (DSR), (2) nest daily survival rate conditional upon adult survival (“conditional nest DSR”), (3) overall brood survival rate conditional upon adult survival (“conditional brood survival rate”), and (4) overall survival rate of individual ducklings conditional upon at least partial brood survival (“conditional duckling survival rate”) within the base model parameterization until we reached specific targets for duckling production. These targets were 1.05 and 1.11 total ducklings/adult female for the medium and high production scenarios, respectively. We applied the sets of breeding season survival parameters comprising each of the 3 production scenarios to all of the 640 unique MMEM simulation outputs, resulting in 1920 (3 × 640) possible sets of starting states for a given run of the reproduction model (Figure 2).

The second issue with limiting the number of reproduction model runs stemmed from the variability in outputs in both the MMEM and the reproduction model due to stochastic elements. The reproduction model was replicated 200 times for each of the 1920 sets of starting states, resulting in 384,000 total runs of the reproduction model (Figure 2).

To conduct those 384,000 runs, we partitioned the runs into 32 groups of 12,000 runs. Each group was run in parallel instance of a statistical computing environment (R Version 4.5.0, R Core Team 2025). We made results reproducible by setting a pseudo‐random number generator seed equal to 73*x*, where 73 was an arbitrary number, and *x* was a number denoting a group of runs; it ranged from 1 to 32 (‘set. seed’ function in R, R Core Team 2025). We delivered the model outputs and code as a U.S. Geological Survey data release (West et al. 2025).

### 2.4 Post‐Modeling Summary and Analysis

To interpret results across this large simulation set, our post‐modeling data summary and analysis had three proximate goals. The first proximate goal was to summarize the results of the low production scenario (i.e., the base reproduction model), along with the medium and high production scenarios, in the absence of oiling; methods and results specific to those summaries are contained in Appendix E. The second proximate goal was to summarize and analyze the differences in model outputs relevant to population vital rates (e.g., duckling production and adult survival) between simulations with either trace or light oiling and the corresponding replicate unoiled baseline simulations. Our third proximate goal involved summary and analysis to support the identification of sublethal effects of oiling on reproduction. The second and third proximate goals were accomplished with second set of summaries and analyses that supported our overarching primary goal of estimating how timing of oil spills and intensity of oiling affect reproductive output in mallards. For all sets of data summaries, all results were rounded to three significant digits up to a maximum of three decimal places.

All data summaries dealt with at least six output variables: (1) total duckling production, (2) estimated population growth rate, (3) per‐adult duckling production rate, (4) adult survival rate, (5) nests per adult, and (6) mean clutch size. Total duckling production, that is, the total number of ducklings produced by the simulated adult mallard population, was an estimate of raw reproductive output, and it was especially useful for comparing oiled vs. unoiled simulations. Estimated population growth rate only applied to the female population, was calculated using Equation D1 in Appendix D for the prior non‐breeding season and modeled breeding season, and assumed a nonbreeding season survival rate of 0.800 prior to the oil spill (based on Hoekman et al. 2002). Per‐adult duckling production rate was total duckling production divided by the number of adult female mallards that arrived at the breeding grounds. Adult survival rate, that is, the number of adult female mallards that survived the breeding season divided by the number of adult female mallards that arrived on the breeding grounds, was used as an important population demographic rate that could be directly and indirectly affected by oiling via body condition effects. Nests per adult was the number of nests (first nests + renesting attempts) created by the simulated population divided by the number of adult female mallards that arrived at the breeding grounds. The denominator for mean clutch size was the number of nests created during the reproduction model simulation.

Instead of directly reporting summary statistics for the trace and light oiling levels, we instead based our summaries on the differences between unoiled baseline simulations and corresponding simulations involving trace or light oiling. We used this difference‐based approach because NRDAs that quantify direct avian mortality and reproductive losses are concerned with the number of birds lost (Zafonte and Hampton 2005; for example, Deepwater Horizon NRDA Trustees 2016, USFWS et al. 2012, Sperduto et al. 2003). We summarize both raw differences and percent changes, using the former for comparisons. Within each combination of MMEM starting states (80 sets per oil spill timing, 160 total) and production scenarios (three levels, Figure 2), we created 200 randomly assigned groupings of reproduction model runs, where each run grouping contained one model run from the unoiled baseline and each combination of oiling level × oiling event. Each reproduction model run was assigned to exactly one grouping. For the January events, each run grouping contained five reproduction model runs: one for each of the unoiled baseline, trace oiling × no migration delay, trace oiling × delayed migration, light oiling × no migration delay, and light oiling × delayed migration. For the March event, each run grouping contained three reproduction model runs, one the unoiled baseline, and one for each oiling level. For a given grouping, we subtracted each output variable's value for each oiling level × oiling event combination from the value for the unoiled baseline. Once we calculated those differences, the summarization process was similar to the one we used for the unoiled simulations. First, we calculated the mean value for each difference across all 200 run groupings for each of the 1440 combinations of MMEM starting state (80 for a given spill) × production scenario (3) × oiling level (2) × oiling event (3 across spills; Figure 2). Next, we calculated the median and [0.025, 0.975] interquantile range (95% IQR) for those mean differences across 80 MMEM replicates for each of the 18 combinations of production scenario (3) × oiling level (2) × oiling event (3 across spills).

We interpreted pairs of output variable estimates with non‐overlapping 95% IQRs as different from each other; similar methods have previously been applied when comparing simulation‐based magnitude of effects for bird populations in the context of oil spills (Zafonte and Haptom 2005, Bird and Wildlife Technical Working Group 2007). We performed pairwise comparisons of output variables using these 95% IQRs and used lettering to group pairs with overlapping 95% IQRs (‘multcompLetters’ function of R package ‘multcompView’ version 0.1–1, Graves et al. 2024). We further condensed our data summaries for a given output variable if we determined that production scenario and/or oiling level were factors that were unlikely to meaningfully affect the differences between oiled and unoiled conditions. We made the decision to condense the data summary to a single production scenario if the following two conditions were met: (1) For every oiling event, all production scenarios had overlapping 95% IQRs for each oiling level; (2) pairwise difference patterns across oiling events were identical for across oiling events for each production scenario. Because all output variables met these criteria, we only reported changes between oiled and unoiled simulations for the low production scenario and excluded results for the medium and high production scenarios. This exclusion meant we only reported results based on 128,000 simulations instead of the full set of 384,000; see West et al. (2025) for raw data from all simulations. We made the decision to further condense the data summary to the level of oiling event (i.e., across oiling levels within a single production scenario) if the following two conditions were met: (1) For every oiling event, all production scenario × oiling level combinations had overlapping 95% IQRs; (2) Pairwise difference patterns across oiling events were identical for both oiling levels across oiling events for each production scenario. In all cases where we condense data summaries, we only report data from the low production scenario, as that category used default model parameterization based on Johnson et al. (1987) and Arnold et al. (2010). In cases where we condense data across oiling levels, we aggregated data for each oiling event across MMEMs replicates and oiling level for the low production scenario and calculated one median and 95% CI per oiling event.

## 3 Results

Oil exposure consistently reduced reproductive output across most combinations of spill timing and oiling level. The clearest and most uniform response was a decline in total duckling production, with all scenarios showing decreases and all 95% IQRs excluding zero (Figure 3). These losses were generally larger under light than trace oiling for the January events, whereas differences between oiling levels were not evident for the March event (Figure 3). Estimated population growth rate also declined for most oiling scenarios, with evidence for reductions in five of six oiling‐level × event combinations (Figure 4). For the January spills, light oiling produced larger declines than trace oiling, though estimates for light oiling were more uncertain. Differences among spill timings were not evident within oiling levels (Figure 4).

**FIGURE 3:**
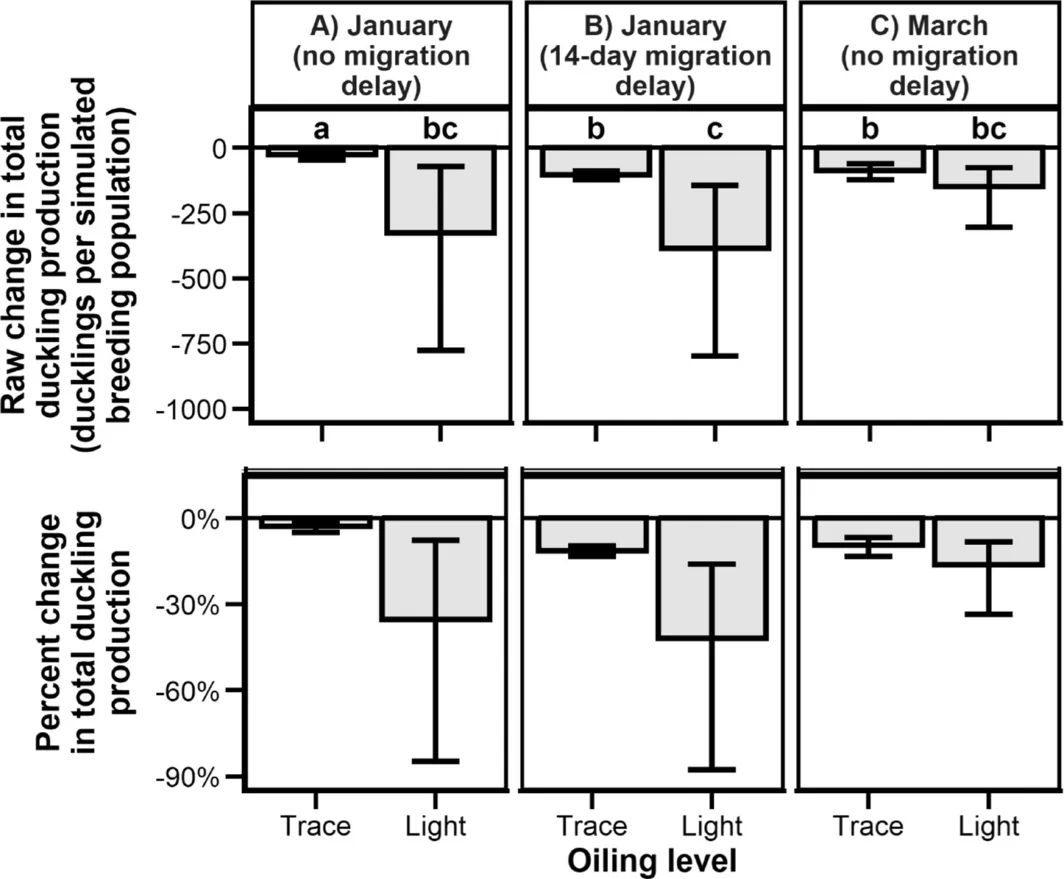
Predicted changes in total duckling production between unoiled and oiled simulated populations of adult female that experienced a simulated oil spill on the wintering grounds. Results are based on 128,000 single‐year breeding season simulations that started with a population of up to 1000 adult female mallards; that number could be reduced by mortality caused by the wintering ground oil spill. The month the spill occurred is indicated in the headings, as is the presence or absence of a delay in departing the wintering grounds. “Adult female” refers to after second year individuals; yearling females were not included in the model. Oiling levels were unoiled (reference condition; not shown), trace (≤ 5% of feather area covered), and light (6%–20% of feather area covered). There were 80 non‐identical replicate starting conditions, and each of those starting conditions was simulated 200 times per oiling level. Values were summarized in a hierarchical manner. First, we calculated mean differences between unoiled and oiled simulations across groups of 200 simulations with identical starting conditions. Next, we used those means to calculate and report median (gray bar) and [0.025, 0.975] quantiles (error bars) across the 80 non‐identical replicate starting conditions. Different letters above bars indicate non‐overlapping [0.025, 0.975] quantiles for raw changes.

**FIGURE 4:**
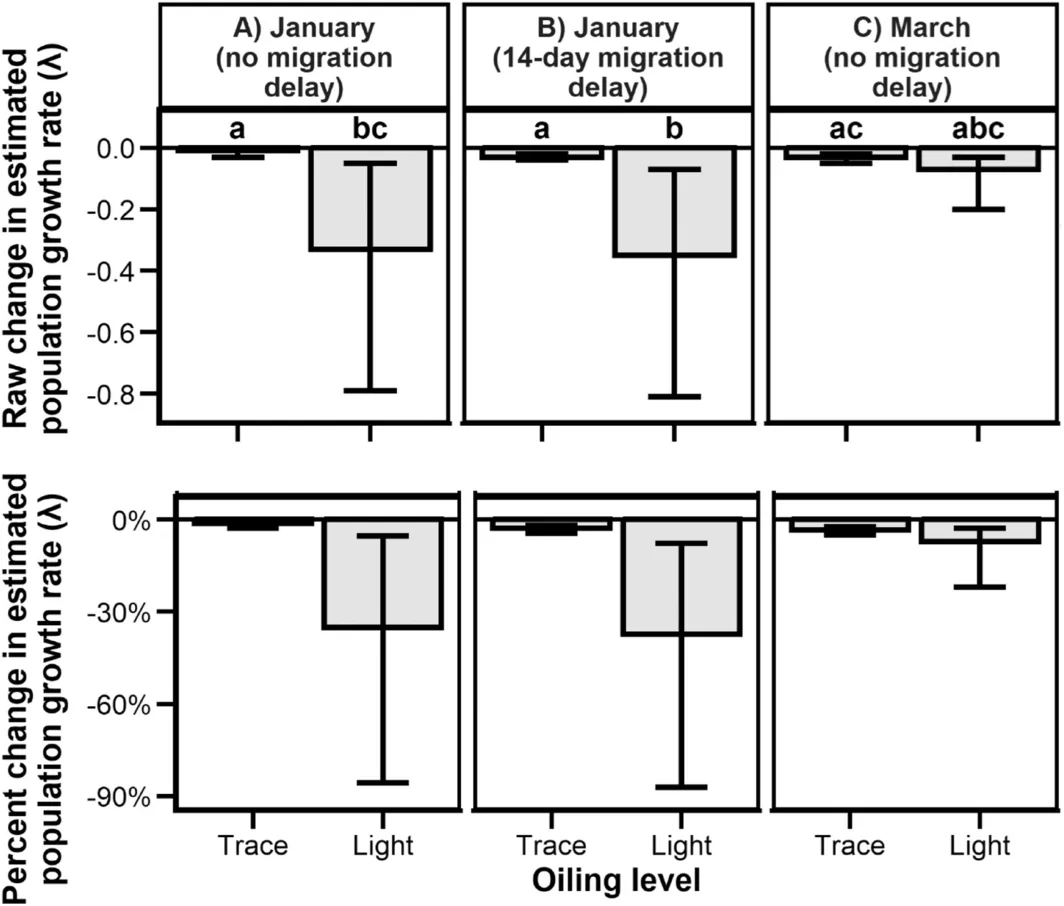
Predicted changes in estimated population growth rates (*λ*) between unoiled and oiled simulated populations of adult female mallards that experienced a simulated oil spill on the wintering grounds. Results are based on 128,000 single‐year breeding season simulations that started with a population of up to 1000 adult female mallards; that number could be reduced by mortality caused by the wintering ground oil spill. The month the spill occurred is indicated in the headings, as is the presence or absence of a delay in departing the wintering grounds. “Adult female” refers to after second year individuals; yearling females were not included in the model. Oiling levels were unoiled (reference condition; not shown), trace (≤ 5% of feather area covered), and light (6% to 20% of feather area covered). There were 80 non‐identical replicate starting conditions, and each of those starting conditions was simulated 200 times per oiling level. Values were summarized in a hierarchical manner. First, we calculated mean differences between unoiled and oiled simulations across groups of 200 simulations with identical starting conditions. Next, we used those means to calculate and report median (gray bar) and [0.025, 0.975] quantiles (error bars) across the 80 non‐identical replicate starting conditions. Different letters above bars indicate non‐overlapping [0.025, 0.975] quantiles for raw changes. Population growth rate was estimated for the modeled breeding season and the prior non‐breeding season and assumed a pre‐oil spill non‐breeding survival rate of 0.800 (based on Hoekman et al. 2002).

For the remaining four variables, per‐adult duckling production rate, adult survival, nests per adult, and mean clutch size, we summarized data to the level of oiling event. Per‐adult duckling production rate decreased for the January delayed‐migration and March events, whereas the January no‐delay event showed no clear change (Table 1). Adult survival showed a different pattern from the duckling production and population growth variables: survival increased for the January delayed‐migration and March events (Table 1), consistent with reduced time spent incubating in scenarios that suppressed nesting activity. There was no evidence of change in adult survival for the January no‐delay event (Table 1). Similarly, nests per adult decreased for both the January delayed‐migration and March events, whereas the January no‐delay event showed no clear change (Table 1). Mean clutch size was largely unchanged except for a decrease in the January delayed‐migration event and a small increase for the March event (Table 1), reflecting shifts in later timing and reduced frequency of renesting, respectively. Consistent with general patterns, for the January no migration delay event, there was no evidence of a change in mean clutch size (Table 1).

**TABLE 1:** Predicted changes in per‐adult‐female duckling production rate, adult female survival rate, nests per adult, and mean clutch size between unoiled and oiled simulated populations of adult female mallards that experienced a simulated oil spill on the wintering grounds.

Variable	Change (unoiled to oiled)			Direction
	Event	Raw	Percent	
Per‐adult‐female duckling	January, no migration delay	−0.02 [−0.04, 0.04]	−2.1% [−3.9%, 4.0%]	N
Production rate	January, delayed migration	−0.11 [−0.13, −0.06]	−10.8% [−12.4%, −6.1%]	−
	March	−0.11 [−0.21, −0.07]	−10.9% [−20.0%, −6.9%]	−
Adult female survival rate	January, no migration delay	0.001 [−0.003, 0.004]	0.08% [−0.47%, 0.53%]	N
	January, delayed migration	0.019 [0.015, 0.024]	2.66% [2.05%, 3.41%]	+
	March	0.006 [0.002, 0.013]	0.81% [0.33%, 1.75%]	+
Nests per adult	January, no migration delay	−0.05 [−0.08, 0.09]	−2.2% [−3.4%, 4.0%]	N
	January, delayed migration	−0.21 [−0.23, −0.12]	−9.5% [−10.2%, −5.2%]	−
	March	−0.24 [−0.44, −0.15]	−10.6% [−19.2%, −6.8%]	−
Mean clutch size	January, no migration delay	0.01 [−0.01, 0.03]	0.1% [−0.1%, 0.3%]	N
	January, delayed migration	−0.65 [−0.66, −0.58]	−7.0% [−7.2%, −6.3%]	−
	March	0.07 [0.04, 0.16]	0.8% [0.4%, 1.8%]	+

*Note:* Results are based on 128,000 single‐year breeding season simulations that started with a population of up to 1000 adult female mallards; that number could be reduced by mortality caused by the wintering ground oil spill. The month the spill occurred is indicated in the headings, as is the presence or absence of a delay in departing the wintering grounds. “Adult female” refers to after second year individuals; yearling females were not included in the model. For “Direction,” *N* = no evidence of change, + = increase from unoiled to oiled, − = decrease. Oiling levels in simulations were unoiled, trace (≤ 5% of feather area covered), and light (6% to 20% of feather area covered). There were 80 non‐identical replicate starting conditions, and each of those starting conditions was simulated 200 times per oiling level. Values were summarized in a hierarchical manner. First, we calculated mean differences between unoiled and oiled simulations across groups of 200 simulations with identical starting conditions. Next, we used those means to calculate and report median and [0.025, 0.975] quantiles for raw and percent changes across the 80 non‐identical replicate starting conditions. We report median and quantiles calculated across both trace and light oiling levels (i.e., 160 oiling simulations per median; 2 oiling levels × 80 starting conditions).

Together, these results show that the dominant carryover effects of oil exposure arise through reductions in duckling production and per‐adult reproductive output, with spill timing and arrival condition shaping the magnitude of losses.

## 4 Discussion

Our results show that oil exposure during winter and migration, even at trace levels, consistently reduced reproductive output across populations of adult female mallards, with declines in total and per‐adult duckling production emerging as the most pronounced carryover effects. Total duckling production declined in all six simulated oil spill cases we examined, and it was the product of the complement of non‐breeding season oil spill mortality rate (i.e., survival rate) from the MMEM and per‐adult duckling production in the reproduction model. Uncertainty was greatest under the light oiling scenario, largely reflecting the high variability in MMEM derived estimates of oil‐related adult mortality (West et al. 2026a). The MMEM is the primary source of this uncertainty, as evidenced by the much smaller variability in per‐adult duckling production rates that do not incorporate oil‐induced adult mortality. Reducing uncertainty in total duckling production estimates would require more precise mortality estimates for oil spills occurring on the wintering grounds. For the January events, MMEM‐derived adult mortality from the oil spill was also the driver of differences among oiling levels in terms of magnitude of changes in total duckling production, as we did not find evidence of differences between oiling levels for per‐adult production rate within any oiling event.

Per‐adult duckling production rate declined in five of the six simulated oil spill cases we examined. The only case where it did not decline, the light oiling level within the January no migration delay event, was due to a lack of difference in adult arrival body masses in the MMEM (West et al. 2026a) and unaltered migration timing. Within our modeling framework, we considered per‐adult duckling production rate the clearest indicator of sublethal effects on reproduction because it only included adults that arrived on the breeding grounds, and pertained to the ultimate outcome of reproduction, production of surviving offspring. This model‐based finding of sublethal effects on reproduction provides evidence that accounting for sublethal effects, along with the lethal effects typically included in avian population injury assessments (e.g., Deepwater Horizon NRDA Trustees 2016; Bird and Wildlife Technical Working Group 2007; USFWS et al. 2012; Sperduto et al. 2003), could provide a more accurate assessment of foregone avian reproduction in the wake of oil spills.

Regarding the effects of simulated oil spills on estimated population growth rate, the patterns largely mirrored those of two of its component values: oil spill mortality from the MMEM (West et al. 2026a) and total duckling production. Though a third component, adult female breeding season survival rate, was incorporated into the calculation of estimated population growth rate, qualitatively, this incorporation had little apparent effect on the patterns of estimated population growth rate. Despite increases in adult female breeding season survival rate for the January delayed migration and March events, the change in estimated population growth rate was distinctly negative across both oiling levels for those events. The primary apparent contribution of adult female breeding season survival rate was increased variability, though we did not directly quantify this contribution. As an example, trace oiling level for the January no migration delay event did not meet our threshold for evidence of change for estimated population rate, despite there being evidence of a difference for total duckling production and no evidence of differences for oil spill mortality from the MMEM (West et al. 2026a) and total duckling production. Mallard populations tend to be similarly sensitive to changes in nonbreeding and breeding season survival of adult females (Hoekman et al. 2002; Coluccy et al. 2008; Amundson et al. 2013). This pattern is consistent with the much larger mortality imposed during the nonbreeding period in the MMEM, especially for light oiling and January events, compared with the minimal changes in breeding‐season survival estimated within the reproduction model.

Mallards in our simulations renested often, with median unoiled baseline simulations exceeding 2.20 nests per female (Table E1 in Appendix E; note that prefixes on appendix table and figure numbers correspond to the lettering for a given appendix). Oiling reduced the average number of nests produced by modeled female mallards. However, renesting propensity can vary among species and across populations within species (Thompson et al. 2001). Some bird species will not attempt to renest after a nest fails; some species will only attempt to renest once; and other species, like the mallard, may exhibit multiple renesting attempts (e.g., Arnold et al. 2010). Additionally, species that do renest may also only do so at a low frequency. For example, a population of piping plover ( *Charadrius melodus* ) in the northern Great Plains of the United States only renest at a rate of 25% (Swift et al. 2020). Because of these differences among life history strategies, our parameterization of this model specifically for mallards is unlikely to be applicable to species that do not renest frequently. However, reparametrizing this model to match other species' life history traits could make it a valuable tool beyond mallards and species with similar breeding life history.

A surprising finding was the lack of difference among production scenarios in terms of oil spill effects. Previous simulation modeling suggests that populations with higher growth rates experience greater effects following oil spill‐induced mortalities (Zafonte and Hampton 2005). In our case, we did not find differences among production scenarios, leading to the moderate and high production scenarios not meeting our thresholds for data summary (see West et al. 2025 for raw model results). This was especially surprising given the relatively wide range of baseline estimated population growth rates, specifically 0.93 for low to 1.05 for high production scenarios (Table E1 in Appendix E). We hypothesize that the lack of difference could be due to our modeling framework only including a single year; we did not project effects multiple years into the future. We favor the single‐year hypothesis due to the narrower range of mean population growth rates used by Zafonte and Hampton (2005). Averaged across “good” and “bad” years, those rates were 0.99, 1.00, and 1.02 for the low, medium, and high reproductive success scenarios, respectively. However, additional testing would be needed to determine how mortality and sublethal effects predicted by our modeling framework would propagate within a population across multiple years.

Despite updating components of the original Johnson et al. (1987) model and linking the reproduction model to a migration and energetics model, there are other model components that could be considered in future investigations. Our iteration of the reproduction model dealt with only one reproductive age class and sex: adult females. Oiling of yearling females and males could also affect mallard populations. However, beyond direct mortality, we hypothesize such effects would be less impactful than effects on adult females. In the Johnson et al. (1987) model, yearling females have reduced fecundity due to lower body mass and smaller clutch sizes, and they comprise less than half the population. Generally, oil spill‐induced losses of individuals in lower fecundity groups results in smaller population‐level effects compared to loss within higher fecundity groups (Zafonte and Hampton 2005). Additionally, sensitivity analyses by Johnson et al. (1987) indicated that large changes in the yearling‐to‐adult age ratio of female mallards only had modest effects on duckling recruitment. Male mallards do not incubate or provide parental care (Drilling et al. 2020), and North American mallard sex ratios are biased toward males, meaning there is an excess of males in the population (Baldassarre 2014). As such, male mallards are not assumed to limit duckling production and tend not to be included in mallard reproduction and population models (e.g., Johnson et al. 1987; Hoekman et al. 2002; Coluccy et al. 2008; Amundson et al. 2013). Additionally, there is, to the best of our knowledge, no evidence that body condition of male mallards is associated with reproductive success, though plumage quality in males can be correlated with reproductive success (Sheppard et al. 2013). Given that most mallard pairs form before spring departure from the wintering grounds (Drilling et al. 2020), it seems reasonable to assume that most mallard pairs would have both the male and female oiled during a wintering ground oil spill. Even though those factors hypothetically diminish the likelihood that oiling of male mallards could affect population‐level reproductive output, it is possible that other factors, such as disruption to pair bonds or reduced ability to secure breeding territory, could still affect individual mallard pairs. Additionally, consideration of males in terms of effects on population‐scale reproduction would likely be of greater concern for bird species where mating pairs form on breeding grounds and/or males are more involved with incubation and chick‐rearing.

Our modeling framework also did not consider density dependence, a decision we made due to how mallard migratory ecology relates to population‐level breeding site distribution. Adult female mallards in the MMEM, the data source for the reproduction model, start in a single 103,600 ha wintering node and spread across a breeding region comprising 547 nodes of the same area, that is, 56.67 million ha across the southeastern Prairie Pothole Region (West et al. 2026a, 2026b). This dispersal of mallards from a relatively small wintering area across a much larger breeding area is supported by the telemetry observations underpinning the MMEM (Krementz et al. 2011; Teitelbaum et al. 2023) and other similar studies (e.g., Masto et al. 2025). Thus, because a wintering ground oil spill is unlikely to affect a large number of female mallards arriving at the same breeding site, we did not expect population‐level density dependence effects. However, oil spill‐related effects could still interact with density dependence, potentially warranting additional investigation. Both reduced body condition and late arrival to a breeding site could force previously oiled female mallards to select poorer breeding sites. Density dependence‐related effects on avian reproduction after oil spill may also be a greater factor for bird species with more restricted breeding areas, such as island‐nesting seabirds.

Before this model is applied to an oil spill injury assessment, additional factors would need to be considered. First, the likelihood that a breeding‐age female transports oil on their feathers to the breeding grounds should be assessed. This likelihood will probably be higher if the spill happens closer to the breeding grounds in time and space. Any oil that gets on eggs can cause embryotoxicity, potentially resulting in nest failure or unviable ducklings (King et al. 2021). Toxic effects from oiling may also cause behavioral or hormonal changes that could prevent a female from reproducing (King et al. 2021), so quantifying the magnitude, prevalence, and persistence of these toxic effects would benefit the accuracy of estimates. Finally, increased certainty about the degree of migration delays and mortality after oil spills could also improve estimates.

A specific aspect of NRDA where this modeling framework could be applied is in the context of a resource equivalency analysis (REA, Desvousges et al. 2018, Baker et al. 2020). The end goal of REA is to scale restoration after a contamination event, for example, oil spill, so that services lost due to the event are compensated for by restoration‐created gains in services (Desvousges et al. 2018). A REA focuses on a single species or population, often birds, and determines the quantity of lost services provided by that species or population after a spill (Desvousges et al. 2018). Within the context of REA, the type of model we present provides a more complete accounting of lost reproduction than just reproduction lost exclusively through adult mortality. Accounting for reproduction lost only due to adult mortality is an approach that has been taken during multiple oil spills, including, but not limited to, *Deepwater Horizon* (Deepwater Horizon NRDA Trustees 2016), *M/T Athos I* (Bird and Wildlife Technical Working Group 2007), *Bouchard Barge 120* in 2003 (USFWS et al. 2012), and *North Cape* in 1996 (Sperduto et al. 2003). Using or modifying available models, like the combined framework described here and by West et al. (2026a), and incorporating empirical case data to inform the models, lost reproduction could more easily be estimated or accounted for during the injury assessment phase of NRDAs. This combination of modeling and data could improve the breadth and accuracy of injury assessment estimates.

## Author Contributions

**Benjamin M. West:** conceptualization (equal), data curation (lead), investigation (equal), methodology (equal), project administration (supporting), software (lead), visualization (lead), writing – original draft (lead), writing – review and editing (equal). **Mark L. Wildhaber:** conceptualization (equal), formal analysis (supporting), funding acquisition (lead), investigation (equal), methodology (equal), supervision (lead), visualization (supporting), writing – original draft (supporting), writing – review and editing (equal). **Wayne E. Thogmartin:** conceptualization (equal), funding acquisition (supporting), investigation (equal), methodology (equal), project administration (supporting), writing – original draft (supporting), writing – review and editing (equal). **Michael J. Hooper:** conceptualization (equal), funding acquisition (supporting), investigation (equal), project administration (supporting), supervision (supporting), visualization (supporting), writing – review and editing (equal).

## Funding

This work was supported by U.S. Geological Survey. U.S. Department of the Interior.

## Ethics Statement

No animal or human subjects were used in this study. Any use of trade, firm, or product names is for descriptive purposes only and does not imply endorsement by the U.S. Government.

## Conflicts of Interest

The authors declare no conflicts of interest.

## Figures and Tables

**FIGURE A1:**
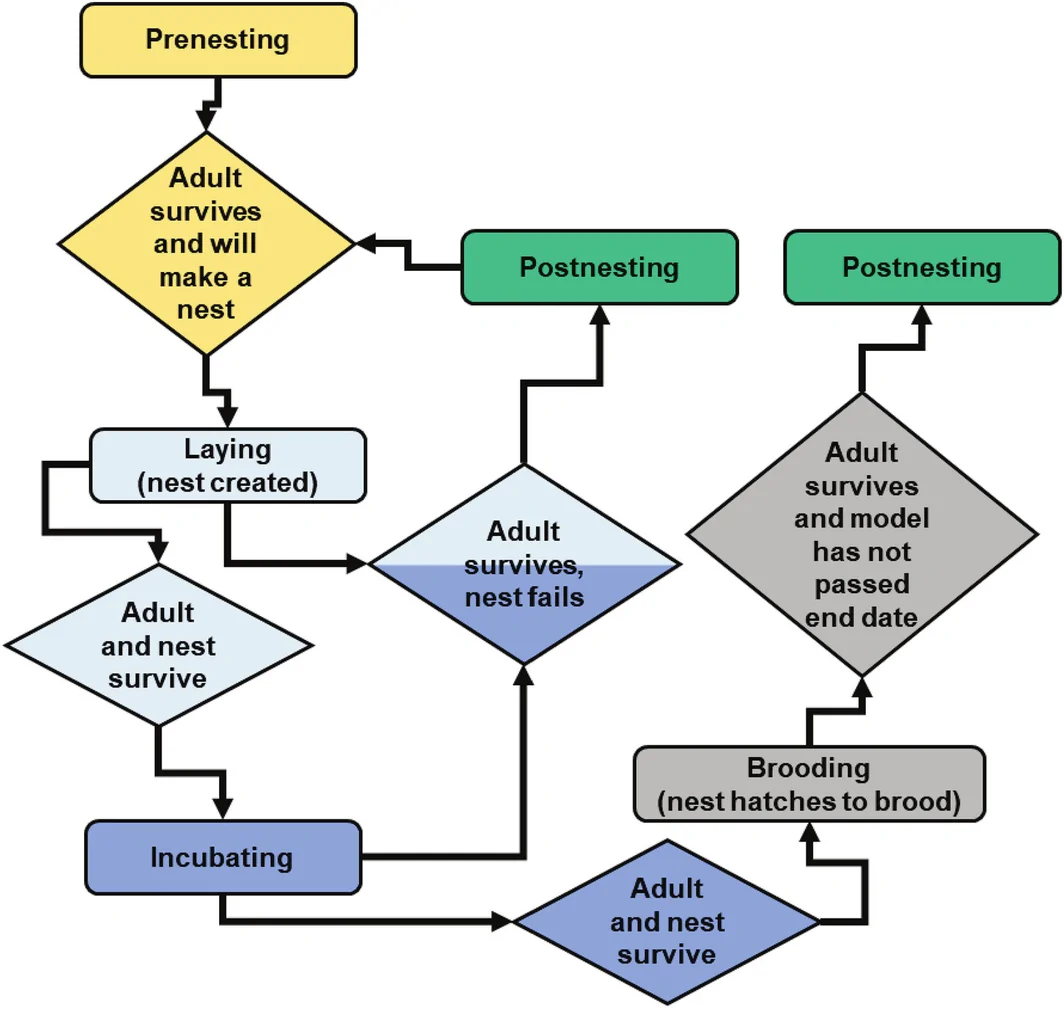
Flow chart demonstrating the “state transition” submodel of a mallard reproduction model. Rounded rectangles are different reproductive states. Rhombuses are conditions that must be met for the submodel to progress to the next state. Colors denote states; yellow is prenesting, lighter blue is laying, darker blue is incubating, gray is brooding, and green is postnesting.

**FIGURE A2:**
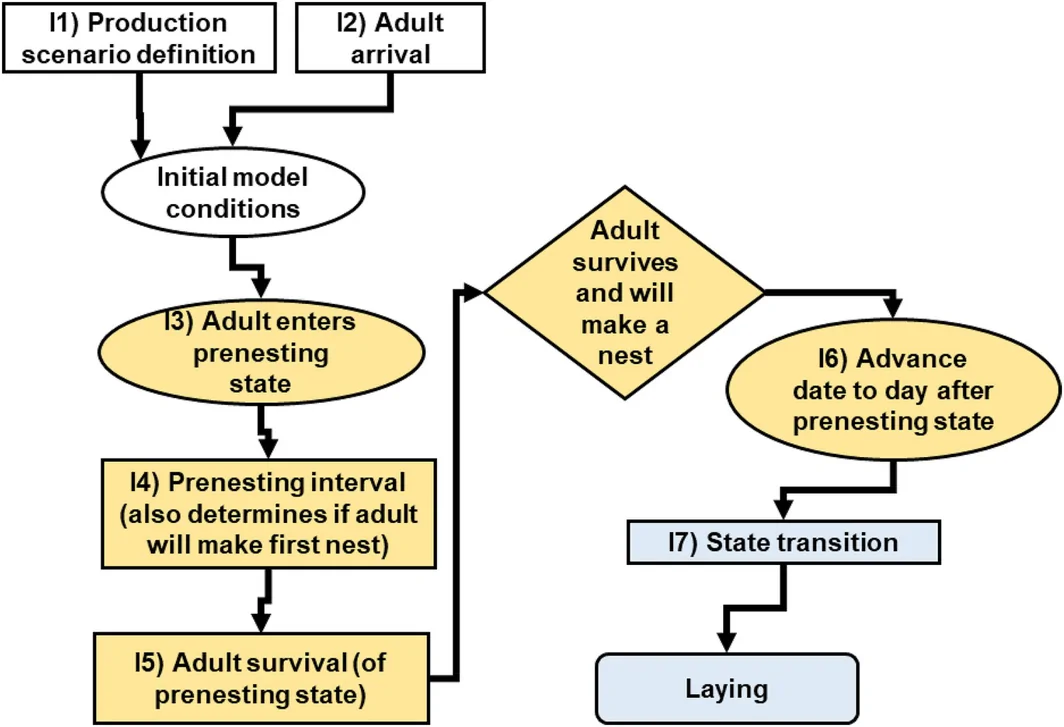
A flowchart detailing initialization steps for a mallard reproduction model. Rounded rectangles is the reproductive state that comes after the initial reproductive state, prenesting. Square‐cornered rectangles are submodels. Rhombuses are conditions that need to be met to progress the next flowchart step. Ovals are scheduling steps that are not classified as submodels. Colors denote states; white is no state assigned, yellow is prenesting, and blue is laying. Prefixes within boxes, such as “I1,” correspond the different steps in 5. Initialization.

**FIGURE A3:**
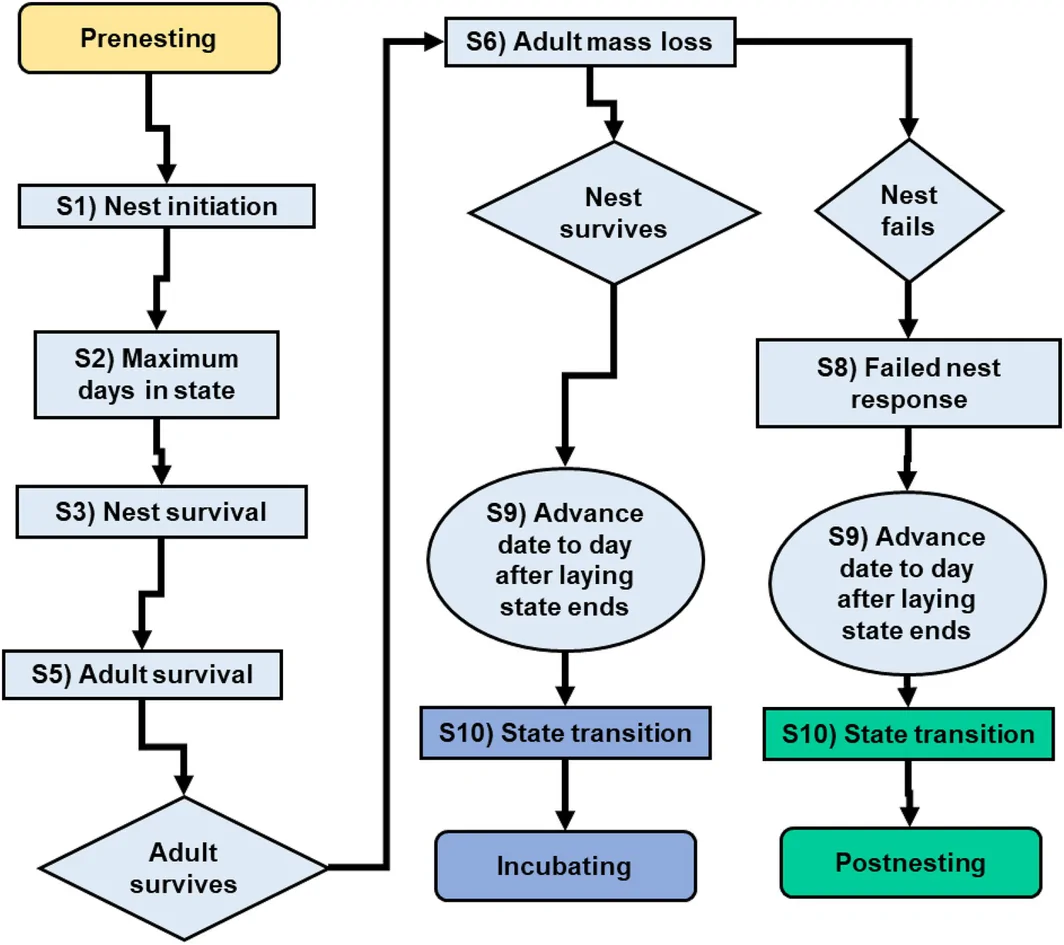
A flowchart detailing process scheduling for the “laying” reproductive state of a mallard reproduction model. Rounded rectangles are other reproductive states that come before or after laying. Square‐cornered rectangles are submodels. Rhombuses are conditions that need to be met to progress the next flowchart step. Ovals are scheduling steps that are not classified as submodels. Colors denote states; yellow is prenesting, lighter blue is laying, darker blue is incubating, and green is postnesting. Prefixes within boxes, such as “S2,” correspond the different scheduling steps in 3. Process overview and scheduling.

**FIGURE A4:**
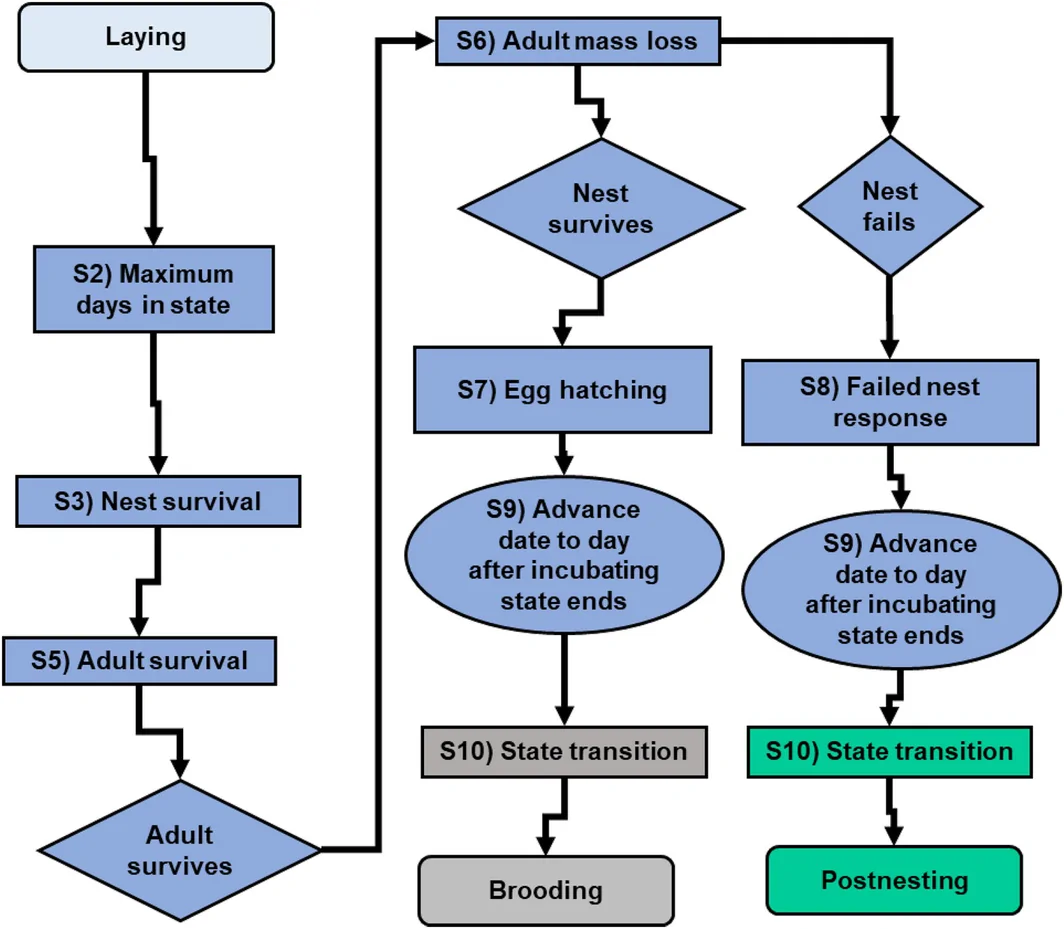
A flowchart detailing process scheduling for the “incubating” reproductive state of a mallard reproduction model. Rounded rectangles are other reproductive states that come before or after incubating. Square‐cornered rectangles are submodels. Rhombuses are conditions that need to be met to progress the next flowchart step. Ovals are scheduling steps that are not classified as submodels. Colors denote states; lighter blue is laying, darker blue is incubating, gray is brooding, and green is postnesting. Prefixes within boxes, such as “S2,” correspond the different scheduling steps in 3. Process overview and scheduling.

**FIGURE A5:**
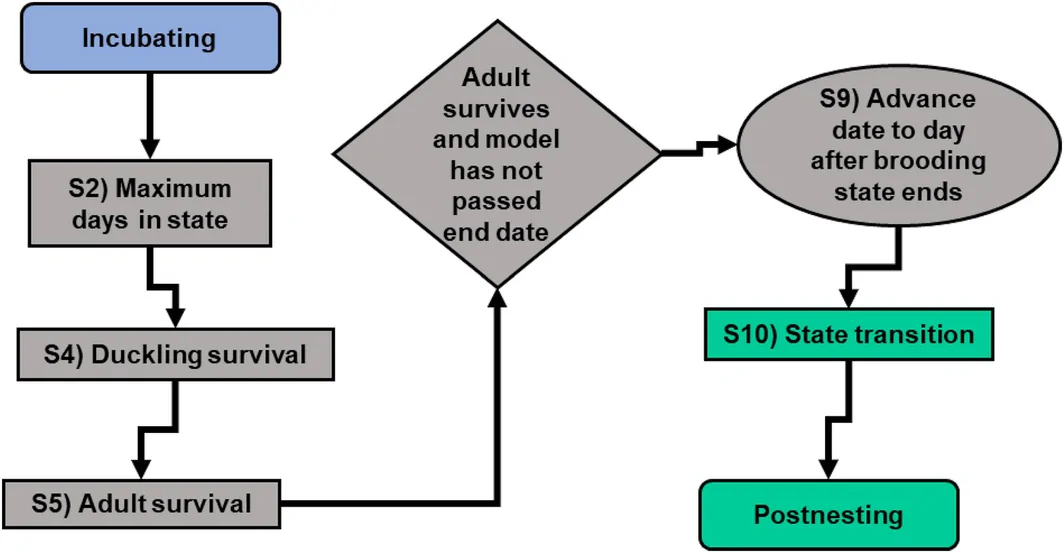
A flowchart detailing process scheduling for the “brooding” reproductive state of a mallard reproduction model. Rounded rectangles are other reproductive states that come before or after brooding. Square‐cornered rectangles are submodels. Rhombuses are conditions that need to be met to progress the next flowchart step. Ovals are scheduling steps that are not classified as submodels. Colors denote states; blue is incubating, gray is brooding, and green is postnesting. Prefixes within boxes, such as “S2,” correspond the different scheduling steps in 3. Process overview and scheduling.

**FIGURE A6:**
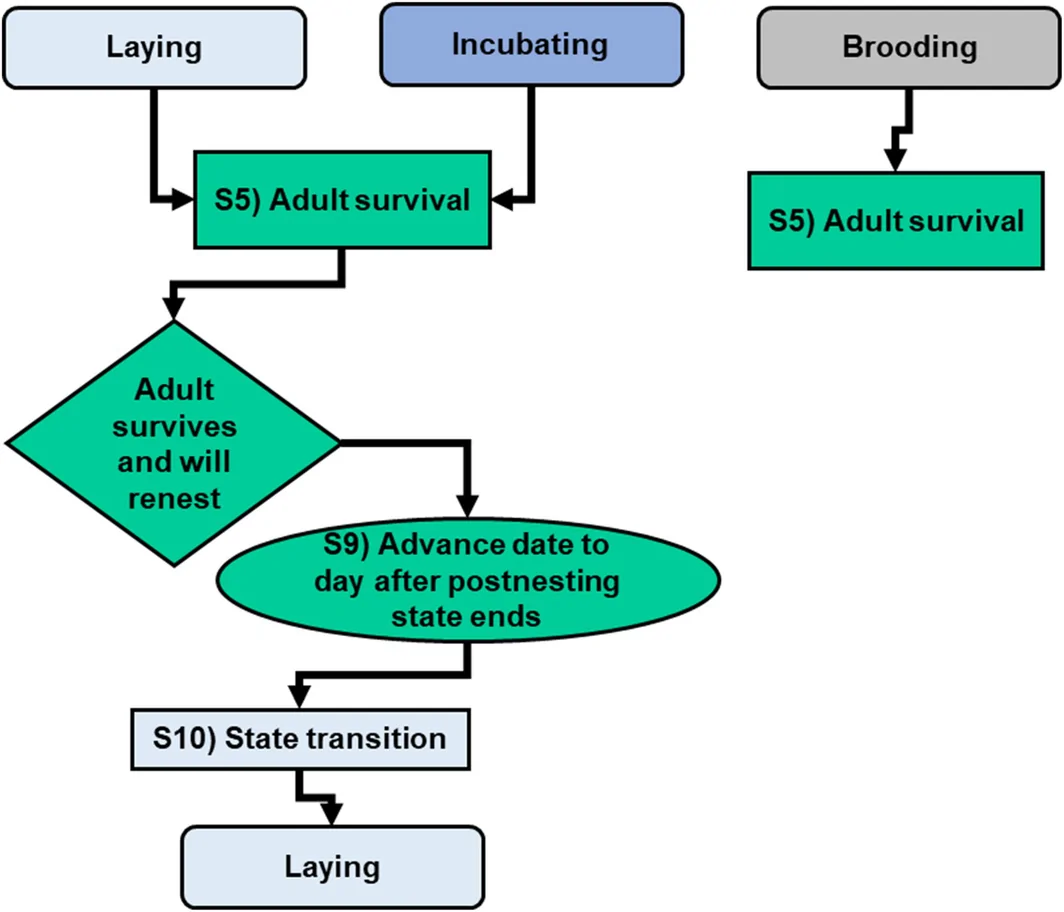
A flowchart detailing process scheduling for the “postnesting” reproductive state of a mallard reproduction model. Rounded rectangles are other reproductive states that come before or after postnesting. Square‐cornered rectangles are submodels. Rhombuses are conditions that need to be met to progress the next flowchart step. Ovals are scheduling steps that are not classified as submodels. Colors denote states; lighter blue is laying, darker blue is incubating, gray is brooding, and green is postnesting. Prefixes within boxes, such as “S2,” correspond the different scheduling steps in 3. Process overview and scheduling.

## Data Availability

Data and code related to this manuscript are freely and publicly available. Model code and the outputs generated by the model are available as a U.S. Geological Survey data release (West et al. 2025; https://doi.org/10.5066/P1KTI8EY). Additional data used to initialize the model can be found within West et al. (2026b; https://doi.org/10.5066/P134V33W).

## Appendix A Model Description

This description is based on the second update to the Overview, Design concepts and Details (ODD) protocol (Grimm et al. 2020). That protocol is designed for agent‐based models; this model lacks certain components of a typical agent‐based model, such as an explicit model world and interaction among agents. Therefore, we only include elements relevant to the model we present, and we rearrange some elements of the original ODD protocol. Code and metadata for the model are available as a U.S. Geological Survey data release (West et al. 2025).

### Purpose

The proximate purpose of the model was to predict how adult survival and offspring recruitment of ducks respond to simulated oil spill effects, namely delayed arrival to the breeding ground and changes in adult body condition, predicted by a companion model (West et al. 2026a). The ultimate purpose was to develop a modeling framework to predict breeding ground survival and reproduction‐related carryover effects of oil spills encountered on the migratory routes of birds. The agents used approximate after‐second‐year female mallards (*Anas platyrhynchos*) and their offspring. In the model, individual mallards arrived at the breeding ground across a distribution of dates with various body masses and entered a breeding sequence that could result in adult mortality, unsuccessful reproduction, or successful recruitment of ducklings.

### Entities, State Variables, and Scales

An adult female mallard was an agent that underwent a sequence of zero to seven breeding attempts over the course of a multi‐month breeding period. Each breeding attempt generated a nest; that nest could fail or hatch into a brood. There was no explicit model world, but it occurred in a generic “breeding area” with parameters that were based on mallards breeding in the Prairie Pothole Region of North America. All dates were ordinal dates.

An adult female mallard had 12 dynamic state variables and 6 static state variables. Nests had 3 dynamic state variables and 8 static state variables. Broods had 0 dynamic state variables and 8 static state variables. All state variables are listed in Table A1.

A simulation included up to 1000 adult mallards and their offspring. Each nest attempt and brood was treated as a single entity, that is, eggs and ducklings were not modeled individually. The model did not have a spatial component. The temporal extent of the model was generally ordinal dates 74 to 236, which corresponds to calendar dates of 15 March to 24 August on non‐leap years; the model could continue past ordinal date 236 if the brood rearing period went past that date (see 3. Process overview and scheduling). The smallest unit of time in the model was a day. Time steps in the model were variable, covering one or more days in which an adult remained in the same state for a given nest number.

### Process Overview, Initialization, and Scheduling

Initialization was moved to this section and is in section 5 in a typical ODD.

#### Process Overview

This model tracks adult female mallards as they transition between up to five reproductive states: prenesting, laying, incubating, brooding, and postnesting (Figure A1). The goal of each adult is to hatch a brood of ducklings and thus reach the brooding state. However, survival of adults, nests, and broods is not guaranteed, and survival rates for adults vary among states. All adults start in the prenesting state, where they decide whether to attempt a first nest. If they make a first nest, they enter the laying state and create a nest. If both the nest and adult survive the laying state, they transition to the incubating state. Like the laying state, both the nest and adult must survive to reach the next state, brooding, where a nest hatches into a brood, satisfying the adult's goal. However, not all broods survive, meaning that not all adults with a brood contribute to the population.

If an adult survives but her offspring perish before the end date of the model, she enters the postnesting state. If offspring are lost during the laying or incubating state, the adult may decide to create another nest. Adults do not create additional nests if they lose a brood.

#### Initialization

Initialization is also demonstrated in Figure A2. The initialization procedure is as follows:

1. Set survival rate modifiers for adults, nests, and broods using submodel “Production scenario definition.”
2. Execute the submodel “Adult arrival”, which introduces a population of adult female mallards (“adults”) into the model and sets each individual's arrival date and body mass.
3. Adults enter the prenesting state.
4. Each adult executes the submodel “Prenesting interval”, which determines if she will make an initial nest and how long she will stay in the prenesting state.
5. Each adult executes submodel “Adult survival” for the prenesting state, determining survival, and if there was mortality, a mortality date.
6. If a given adult survived the prenesting state and will make a first nest, the day was advanced to the day after the end of the prenesting interval
7. If a given adult survived the prenesting state and will make a first nest, the adult executed submodel “State transition.”

#### Scheduling

The prenesting state is part of initialization (Figure A2). We provide flowcharts depicting the schedules for the following states: laying (Figure A3), incubating (Figure A4), brooding (Figure A5), and postnesting (Figure A6). For each individual adult, its associated nests/brood, and combination of state and nest number:

1. If the adult was in the laying state, the adult executed submodel A7.4. “Nest initiation,” which created a nest.
2. If the adult was in the laying, incubating, or brooding state, the adult executed submodel “Maximum days in state,” determining how long it could stay in its current state. This duration was the default length of the current state before any mortality.
3. If a nest was present, it executed submodel “Nest survival,” determining survival and, if it failed, mortality date. Failure also updated the length of the current state for the adult.
4. If a brood was present, it executed submodel “Duckling survival,” determining the final number of ducklings produced by an adult, and if the brood failed, mortality date. Failure also updated the length of the current state for the adult.
5. The adult executed submodel “Adult survival,” determining survival, and if there was mortality, a mortality date. If a nest or brood was present and the adult experiences mortality, the nest or brood experiences total mortality.
6. The adult executed submodel “Adult mass loss,” which updated its current body mass.
7. If a nest was present and survived Steps 3 and 5, it executed submodel “Egg hatching,” which set initial brood size for Step 10.
8. If there was a nest mortality in Step 3, the adult executed submodel “Failed nest response,” which determined if the adult would renest and set the renesting interval.
9. The date was advanced to the day after the end of the current state
10. The adult and nest (if applicable) executed the “State transition” submodel. This submodel advanced the state of any agents present and could allow a nest previously in the “incubating” state to hatch into a brood with size determined by Step 4.

### Design Concepts

#### Basic Principles

To address our ultimate model purpose, developing a modeling framework to predict breeding ground carryover effects oil spills encountered on the migratory routes of birds, we wanted to select a model organism with well‐studied migration and reproduction where relationships between arrival date, adult body mass, and reproductive output were known. To meet these criteria, we chose the mallard as our model organism. Due to their importance as a game species, reproduction of mallards is heavily studied; in North America, many studies on mallard reproduction originate in the Prairie Pothole Region (reviewed by Baldassarre 2014). This concentration of studies makes mallards in this region an ideal system to model.

We chose to base our modeling effort on an existing mallard reproduction model for the Prairie Pothole Region, created by Johnson et al. (1987). We chose this model specifically because it accounts for the effects of date and body mass on mallard reproduction. These components were important to include because migration delays and reduced body mass upon arrival at the breeding grounds are possible consequences of environmental disturbances such as oil spills (Henkel et al. 2012; West et al. 2022). To further incorporate body mass and date effects, we replaced the adult survival component of the Johnson et al. (1987) model with equations developed by Arnold et al. (2012). Furthermore, we used the results of a companion mallard migration and energetics model incorporating oil spills (West et al. 2026a, 2026b) as the inputs for this reproduction model. Additional changes made to the Johnson et al. (1987) framework are detailed in the “Submodels” section of this appendix.

#### Emergence

The number of nest attempts that each adult female makes is an emergent property of the model.

#### Adaptation

The primary adaptive behaviors in this model are the decisions of adult females to initiate each of their nests.

#### Objectives

The objectives of an adult female are to (1) survive and (2) hatch a nest into a brood of ducklings. Nests and broods do not have explicit objectives.

#### Learning

This model does not incorporate learning.

#### Prediction

By making decisions about renesting attempts, mallards implicitly predict that subsequent nesting attempts may or may not be successful.

#### Sensing

Adult mallards can sense directly nest failures and the number of nests they previously created. There is an implied sensation of their current state, evidenced by different mortality rates for different states.

#### Interaction

This model does not incorporate interactions among individuals.

#### Stochasticity

Stochasticity in this model was implemented as random sampling from distributions for a variety of outcomes. Specifics of this random sampling are described in the “Submodels” section of this appendix; submodels are listed in parentheses. The following values were randomly sampled:

- Whether an adult would create a first nest (Prenesting interval)
- Whether an adult would renest after a nest failure (A7.11. Failed nest response)
- Daily survival of adults and nests (A7.6. Nest survival and A7.9. Adult survival)
- Whether or not an entire brood failed (A7.8. Duckling survival)
- The number of surviving ducklings in a brood (A7.8. Duckling survival)

#### Collectives

Nests are collectives of eggs, and broods are collectives of ducklings. Mortality events can affect entire nests or entire broods.

#### Observation

We observed the model at the level of a simulation, that is, a summary across the simulated population of up to 1000 adult females and any offspring associated with those adults. We recorded the following values:

- Number of adult females at the start of a simulation
- Number of adult females that died while in each of the five states
- Total number of days adults spent alive in each of the five states.
- Overall proportion of adults that attempted at least one nest.
- Number of nests attempted across all adults
- The proportion of adults that survived the prenesting period and attempted a first nest
- Condition renesting proportion, that is, the proportion of adults that had failed first nest, survived the initial postnesting period, and attempted a second nest.
- Mean clutch size of all attempted nests.
- Overall nest survival rate
- Survival rate for an individual duckling (includes total brood failures)
- Mean number of ducklings produced per adult.
- The number of adult females that hatched a nest
- The number of adult females that fledged at least one duckling.

### Initialization

See A3. Process overview, initialization, and scheduling.

### Input Data

This model uses two input files: the results of a mallard migration and energetics model (West et al. 2026b) and a key file used to access those results in piecemeal fashion. The specific input file is named “oil_mod_daily_results.csv” and can be found within West et al. (2026b). The key columns used within model results are adult female body mass and arrival date. The latter file can be found within West et al. (2025).

### Submodels

The state variables used in each submodel are outlined in Table A1.

#### Production Scenario Definition

This submodel sets modifiers to each of adult, nest, and duckling survival, if applicable. The details of how production scenarios were defined and implemented are contained in Appendix D. The same production scenario applied to an entire simulated breeding population of up to 1000 female mallards, and modifiers were constant across time and individuals within a simulation.

#### Adult Arrival

This submodel superseded the Johnson et al. (1987) arrival date and initial body mass submodels. This submodel simply extracted a specified set of 1000 female mallards from a data set detailing arrival dates and initial body masses upon arriving at breeding sites after a wintering and spring migration period (West et al. 2026b). A given set of 1000 female mallards was identified using a “re‐simulation ID” (West et al. 2026b). Any mallards that experienced mortality prior to arriving at their breeding sites were censored.

#### Prenesting Interval

This submodel determined if an adult would attempt a first nest and the date of first nest initiation. The adult enters the “prenesting” state at the start of this submodel. The function for probability of making a first nest was adapted from Devries et al. (2008). Devries et al. (2008) relate probability of making a first nest to a principal‐components analysis‐based body condition index (BCI) that was derived using the natural logarithm (ln) of body mass. Within Devries et al. (2008), Figures 2 and 3, and 5 have x‐axes centered on a BCI of 0 with minimum and maximum values of −250 and 250. We assumed that a BCI of 0 was equivalent to ln(mean body mass), 7.08 (~ln(1193 g)) in our model (rounding to three significant digits intentional), and that −250 and 250 BCI were equivalent to 6.84 (~ln(934 g)) and 7.32 (~ln(1510 g)), respectively, an interval that was symmetrical on a logarithmic scale and approximately bounded by the minimum and maximum body masses for the default settings of our model. With this approximate conversion, we used the after‐second‐year female curve in figure 2 in Devries et al. (2008) to extract x‐y coordinates, convert BCI to grams of body mass, and fit a quadratic equation to those points (Table A2). Once we obtained a probability of a first nest, we used that as a probability of success to randomly sample an outcome from a Bernoulli distribution.

If an adult made a first nest, we calculated the interval between initial arrival and initiation date of that nest. We based this relationship on figure 3 in Devries et al. (2008), which has x‐axis values of BCI (described above) and y‐axis values of relative nest initiation date. Relative nest initiation date was not directly tied to arrival date in Devries et al. (2008), but it was instead the number of days after the first initiated at a site. For mallards observed in North Dakota over multiple decades, the difference between median arrival date and first nest initiation was 20 days (Hammond and Johnson 1984). Therefore, we assumed an adult female mallard with a BCI of 0 would breed 20 days after arrival. In figure 3 of Devries et al. (2008), an after‐second‐year mallard had an average relative nest initiation date of 15.5 days, so we converted relative nest initiation date to days after arrival by adding 4.5 days. With this conversion and the previous BCI conversion, we used the after‐second‐year female curve in figure 3 in Devries et al. (2008) to extract x‐y coordinates, converted the coordinates, and fit a quadratic equation to those points (Table A3). We rounded results of that equation to the nearest integer and added it to arrival date to get the date of first nest initiation. On that date, the adult transitioned to the laying state.

If an adult did not initiate a nest, it stayed in the prenesting state until the end of the simulation.

#### Nest Initiation

This submodel creates a nest and assigns a clutch size to that nest. Clutch size was a deterministic function of ordinal date. Specifically, it was 17.51 − 0.0631 × (ordinal date) rounded up to the nearest whole number (Cowardin et al. 1985; Johnson et al. 1987). This submodel also assigns the number of previous nests to the current nest, increments the cumulative number of nests the adult has initiated by one, and sets eggs laid in last nesting attempt to the current clutch size.

#### Maximum Days in State

The maximum number of days in a state varied by state; maximum days in a state for prenesting and postnesting are handled by the “prenesting interval” and “failed nest response” submodels, respectively.

- Laying: Maximum days in the laying state were the same as clutch size, as mallards lay one egg per day (Drilling et al. 2020). Clutch size was determined by the “nest initiation” submodel.
- Incubating: Maximum days in the incubating state was randomly sampled on a per‐nest basis from a truncated normal distribution with mean = 26, standard deviation = 2.6, minimum = 20, maximum = 31. The mean was the incubation period used by Johnson et al. (1987), the standard deviation was the midpoint between the standard deviations reported by Feldheim (1997) and Wells‐Berlin et al. (2005), and the range was from Feldheim (1997). Sampled values were rounded to the nearest integer.
- Brooding: Maximum days in the brooding state were 51 days, a rounded equivalent of the mean of 50.7 days listed by Ball et al. (1975).

#### Nest Survival

This submodel had three phases: (1) calculation of daily survival rate, (2) sampling of daily survival outcomes, and (3) nest mortality and hatching handling. Like Johnson et al. (1987), we assume constant underlying nest survival. However, different from Johnson et al. (1987), we treat adult mortality as a separate possible cause of nest mortality, handled by the “adult survival” submodel. The nest mortality calculated by this submodel is independent of adult mortality.

For Phase 1, daily nest survival rates were calculated empirically by running the model until the desired overall nest survival was reached. The daily nest survival rate to reach approximately 0.119 overall nest survival, the standard nest survival given by Johnson et al. (1987), was 0.9499.

For Phase 2, survival outcomes were randomly sampled on each day in the current state (laying or incubating) from a Bernoulli distribution with success probability = (daily nest survival rate). If any random samples returned a value of zero, the nest failed, and submodel 3 was initiated.

For Phase 3, the nest mortality date was the first day in the current state where Phase 2 returned a value of zero. This mortality date was the last day in the state for the adult before it transitioned to the postnesting state; see the “state transition” submodel. The state of nest failure and nest failure date were recorded. If the nest was in the incubating state and survived the entire state, it progressed to the egg hatching model.

#### Egg Hatching

A hatching date was assigned as the day after the incubating state ended. That hatching date also applied to the brood that hatched from the nest. The number of eggs that hatched into ducklings was randomly sampled from a binomial distribution with size equal to clutch size and a hatching probability of 0.91 (Johnson et al. 1992, Hoekman et al. 2002; rounding to two decimal places originates from cited sources). To ensure all nests that hatched were successful, the minimum number of eggs that could hatch was one; any sampled values of zero were converted to one.

#### Duckling Survival

Like Johnson et al. (1987), duckling survival was handled in two phases: (1) determine if there was entire loss of a brood, and (2) determine the number of surviving ducklings if the brood survived. We added an additional third phase for broods with total mortality to determine their mortality date.

For Phase 1, survival of at least one duckling in a brood was sampled from Bernoulli distribution. The survival probability for this distribution was equal to 1 – 0.26 × (mortality rate multiplier); see Appendix D for mortality rate multiplier values and derivation. A value of 0.26 was the probability of complete brood loss in a default scenario; it was based on the brood survival rate of 0.74 used by Johnson et al. (1987).

If at least one duckling survived in Phase 1, Phase 2 determined the number of surviving ducklings. Johnson et al. (1987) used a proportional multiplier of 0.54 for the surviving number of ducklings in a brood; we instead randomly sampled the number of remaining ducklings based on a mean survival rate. Mean individual survival rate was calculated as 1 – 0.46 × (mortality rate multiplier). A value of 0.46 was the individual duckling mortality rate in a default scenario; it was based on the duckling survival rate of 0.54 used by Johnson et al. (1987). Using the mean individual survival rate, we calculated a target number of surviving ducklings as (mean individual survival rate) × 9 × 9/8.99, where 8.99 is the mean brood size before mortality (empirically based on model outputs), and 9 is 8.99 rounded to the nearest whole number. We set the mean of a binomial sampling distribution as (target number of surviving ducklings) – 1, the target number of ducklings surviving minus the one surviving duckling required to be alive to prevent a total brood failure. We then randomly sampled from a binomial distribution with size = (brood size at hatching) – 1 and success probability of ([target number of surviving ducklings] – 1)/8, where 8 is (rounded mean brood size before mortality) − 1. We added 1 to the sample value to get a final surviving brood size.

If no ducklings survived in Phase 1, Phase 3 randomly sampled a mortality date for the brood. We assumed that 70% of brood mortalities occurred by 2 weeks of age (Ball et al. 1975) and that brood mortality probability followed an exponential probability distribution where x‐values were the number of weeks after hatching. Using these assumptions, we solved for an exponential rate parameter of 0.602. We randomly sampled the mortality week from an exponential distribution with that rate parameter. We converted weeks after hatching to days after hatching by multiplying by 7, and we truncated any sampled values above 50 days after hatching to 50 because a brood that survived 51 days or longer survived (see “maximum days in state” submodel). The final equation was

(S1)
$$d=\min\left(7\times \text{Exponential}\left(\lambda =0.602\right),50\right)$$

where *d* is days after hatching, and *λ* is the exponential rate parameter.

We calculated the date of brood failure from hatching date and days after hatching date, the brooding state stopped on that date, and the adult went to the postnesting state on the day after brood failure and stayed in that state until the end of the simulation.

Note that we treat adult mortality as a separate possible cause of brood mortality; this mortality source is handled by the “adult survival” submodel.

#### Adult Survival

This submodel involved determining daily adult survival probability, sampling a daily survival outcome for each day in a state, and determining the mortality date, if applicable. For each day in a state, daily adult survival probability was calculated using the “Combined Reproductive Periods” submodel in Appendix 1 of Arnold et al. (2012). The predictors of adult survival in this equation are modified ordinal date (ordinal date – 90), female age (second‐year or after‐second‐year), presence of a transmitter attached to the adult, centered and scaled body mass, current state (prenesting, laying, incubating, brooding, postnesting), and days spent in the current state. We set the age term to 2 (after‐second‐year); we set the transmitter term to 0; and we center‐scaled mass using the formula ([arrival body mass]− 1200 grams)/80 grams, where 1200 and 80 grams were mean and standard deviation of initial body mass from Johnson et al. (1987). Daily mortality probability was calculated as 1 – (daily survival probability), and a mortality rate multiplier from Appendix D was applied to this probability if assigned by Submodel “Production scenario definition.” Daily mortality probability was converted back to daily survival probability before random sampling.

Survival outcomes were randomly sampled on each day in the current state from a Bernoulli distribution with success probability = (daily survival probability). If any random samples returned a value of zero, the adult died, as did any associated nests or broods. Mortality date was calculated as the first day in the current state where the daily survival outcome was zero. The adult was no longer simulated after mortality, and the mortality for the adult and any associated nests or broods was recorded.

#### Adult Mass Loss

Adults lost 10 grams of body mass for every day they spent in the laying state, and 2 grams for each day spent in the incubating state. These mass loss rates are directly from Johnson et al. (1987). Adults did not lose body mass in other states.

#### Failed Nest Response

This submodel determines (1) if an adult will renest and (2) when the adult will initiate a new nest.

Renesting probability was based on an equation provided by table 1 of Arnold et al. (2010). The predictors of renesting probability in this equation are calendar year, site, female age, number of previous nest attempts, initiation date of the failed nest, days between initiation of the failed nest and failure date, and adult female body condition prior to initiating any nests. Within the equation, we set female age to 2 (after second year), we set the year and site terms to their average values (−0.305 and −0.510, respectively), and we subtracted 1193 grams (mean body mass for unoiled birds in the input data set, West et al. 2026b) from initial body mass to arrive at an adjusted body mass meant to be equivalent to the grams‐based body condition metric used by Arnold et al. (2010). Arnold et al. (2010) note that raw mass performs similarly to their body condition metric in their equations. After calculation of renesting probability, we randomly sampled the renesting outcome from a Bernoulli distribution using renesting probability as the probability of success.

We added two additional conditions that superseded the sampled renesting outcome. First, if the current mass of the adult after nest failure was less than 900 grams, she would not renest, per Johnson et al. (1987). Second, the total number of nest attempts for an adult would not exceed seven; seven was the maximum number of nesting attempts observed by Arnold et al. (2010).

If an adult renested, we calculated the interval between nest failure and the initiation of the new nest. If the nest failed during the laying stage, we used a previously described equation to calculate the renesting interval (table 2 on p. 217 of Arnold et al. 2010). The predictors of renesting interval in this equation are calendar year, site, female age, initiation date of the failed nest, and days between initiation of the failed nest and failure date. Within the equation, we set the year and site terms to their average values (−0.263 and −0.010, respectively). If the nest failed during the incubation stage, we set renesting interval to 10 days, the median renesting interval for adult female mallards observed by Arnold et al. (2010). Regardless of whether the nest failed during the laying or incubating stage, we set maximum renesting interval to 10 days, as incubation of a fully laid nest represents more energetic investment than laying a nest. At the end of the renesting interval, the adult entered the laying stage. If an adult did not renest, it stayed in the postnesting state until the end of the simulation.

#### State Transition

This submodel was a set of decision rules on which states could transition into other states. It is demonstrated in Figure A1.

- The prenesting state could only transition to one other state, laying, if the “prenesting interval” submodel determined the adult would attempt a nest.
- The laying state could transition to the incubating state if a nest survived the entire laying state, or it could transition to the postnesting state if the nest failed.
- The incubating state could transition to the brooding state if a nest survived the entire incubating state, or it could transition to the postnesting state if the nest failed.
- The brooding state could transition to the postnesting state if the brood became independent before the end of the model or the entire brood was lost.
- The postnesting state could transition to the laying state if the adult decided to renest; this decision comes from the “failed nest response” submodel. An adult that reached the brooding state would never transition back to the laying state from the postnesting state.

### Tables for Appendix A

**TABLE A1:** Descriptions of state variables that applied to entities used in a mallard reproduction model.

Entity	Type	ID	Description	Possible values	Source(s)
*Production scenario definition*					
Adult	Static	AS01	Multiplier applied to adult daily mortality rate	1.000; 0.914; or 0.795	Appendix D of current study
Nest	Static	NS01	Multiplier applied to conditional nest daily mortality rate	1.000; 0.524; or 0.528	Appendix D of current study
Brood	Static	BS01	Multiplier applied to conditional brood and duckling mortality rates	1.000; 0.647; or 0.637	Appendix D of current study
*Adult arrival*					
Adult	Static	AS02	Ordinal date of arrival at the breeding area	Ordinal dates 74–144, inclusive	West et al. 2026b
Adult	Static	AS03	Initial mass upon arriving at the breeding area	500–2000 g	West et al. 2026b
*Prenesting interval*					
Adult	Dynamic	AD01	Breeding state	Prenesting, Laying, Incubating, Brooding, Postnesting	Arnold et al. 2012
Adult	Dynamic	AD02	Date range where an adult can remain in its current state	Any range of ordinal dates between 74 and 236 encompassing 51 days or fewer	—
Adult	Dynamic	AD03	Date of last nest initiation	Ordinal date from 74 to 236, inclusive	—
Adult	Static	AS02	Ordinal date of arrival at the breeding area	Ordinal dates 74–144, inclusive	West et al. 2026b
Adult	Static	AS03	Initial mass upon arriving at the breeding area	500–2000 g	West et al. 2026b
Adult	Static	AS04	Probability of initiating their first nest	Any number between 0 and 1, inclusive	Devries et al. 2008
Adult	Static	AS05	A binary indicator as to if the mallard starts a first nest	0 or 1	—
Nest	Static	NS02	Initiation date	Ordinal date from 74 to 236, inclusive	—
*Nest initiation*					
Adult	Dynamic	AD04	Eggs laid in last nesting attempt	5–13	—
Adult	Dynamic	AD05	Cumulative number of nests initiated	0–7	—
Nest	Static	NS03	Clutch size	5–13	Johnson et al. 1987
Nest	Static	NS04	Number of preceding nests	1–6	—
*Maximum days in state*					
Adult	Dynamic	AD01	Breeding state	Prenesting, Laying, Incubating, Brooding, Postnesting	Arnold et al. 2012
Adult	Dynamic	AD06	Maximum days in state	1–163	—
Nest	Dynamic	ND01	Maximum days in state	5–31	Johnson et al. 1987, Feldheim 1997, Wells‐Berlin et al. 2005
Nest	Static	NS02	Clutch size	5–13	Johnson et al. 1987
Brood	Static	BS02	Maximum days in state	51	Ball et al. 1975
*Nest survival*					
Adult	Dynamic	AD07	State in which an adult's last nest or brood failed	Laying or Incubating	—
Adult	Dynamic	AD08	Date of last nest failure	Ordinal date from 74 to 236, inclusive	—
Nest	Dynamic	ND01	Maximum days in state	5–31	Johnson et al. 1987, Feldheim 1997, Wells‐Berlin et al. 2005
Nest	Dynamic	ND02	Daily survival status	0 or 1	—
Nest	Dynamic	ND03	Status as either being currently laid or incubated	Laying or Incubating	—
Nest	Static	NS01	Multiplier applied to conditional nest daily mortality rate	1.000; 0.524; or 0.528	Appendix D of current study
Nest	Static	NS05	Conditional nest daily survival rate	0.947 or 0.972	Johnson et al. 1987
Nest	Static	NS06	Failure date	Undefined, or an ordinal date from 74 to 236, inclusive	—
*Egg hatching*					
Nest	Static	NS02	Clutch size	5–13	Johnson et al. 1987
Nest	Static	NS07	Individual egg hatching probability	0.91	Johnson et al. 1992, Hoekman et al. 2002
Nest	Static	NS08	Hatch date	Ordinal date from 74 to 236, inclusive	—
Brood	Static	BS03	Hatch date	Ordinal date from 74 to 236, inclusive	—
Brood	Static	BS04	Initial number of ducklings	5–13	—
*Duckling survival*					
Brood	Static	BS01	Multiplier applied to conditional brood and duckling mortality rates	1.000; 0.647; or 0.637	Appendix D of current study
Brood	Static	BS05	Conditional brood survival	0.751, 0.839, or 0.841	Johnson et al. 1987
Brood	Static	BS06	Conditional duckling survival	0.540, 0.702, or 0.707	Johnson et al. 1987
Brood	Static	BS02	Binary overall brood survival	0 or 1	—
Brood	Static	BS04	Initial number of ducklings	5–13	—
Brood	Static	BS07	Number of surviving ducklings	0–13	—
Brood	Static	BS08	Mortality date if the entire brood was lost	Undefined, or an ordinal date from 74 to 236, inclusive	—
*Adult survival*					
Adult	Dynamic	AD08	Daily survival status	0 or 1	—
Adult	Dynamic	AD09	Adult daily survival rate	Any number between 0.000 and 1.000, inclusive	Arnold et al. 2012
Adult	Static	AS01	Multiplier applied to adult daily mortality rate	1.000; 0.914; or 0.795	Appendix D of current study
Adult	Static	AS03	Initial mass upon arriving at the breeding area	1000–1400 g*	West et al. 2026b
Adult	Static	AS06	Mortality date	Undefined, or an ordinal date from 74 to 236, inclusive	—
*Adult mass loss*					
Adult	Dynamic	AD10	Current body mass	Any positive integer (grams)	—
*Failed nest response*					
Adult	Dynamic	AD05	Cumulative number of nests initiated	0 to 7	—
Adult	Dynamic	AD10	Current body mass	Any positive integer (grams)	—
Adult	Dynamic	AD11	Binary indicator as to if an adult will renest after the failure of its current nest	0 or 1	—
Adult	Dynamic	AD12	The amount of time after nest failure that an adult will attempt to initiate a new nest	1 to 10 days	Arnold et al. (2010)
Nest	Static	NS02	Initiation date	Ordinal date from 74 to 236, inclusive	—
Nest	Static	NS06	Failure date	Undefined, or an ordinal date from 74 to 236, inclusive	—

*Note:* Gray rows with italic text signify the submodel a variable was used in. The “Type” column describes if the variable can change at least once during the model (“Dynamic”) or is assigned a set value for an individual (“Static”). The ID column has a unique identifier for each variable, as some variables are repeated in the table due to being used in multiple submodels. A “—” in the “Source(s)” column means the variable is defined within the model and does not directly originate from another source; note that some values may not directly match the original source due to modification made within the modeling framework. Nest and brood mortality and survival rates are conditional upon adult survival; duckling mortality and survival rates are conditional upon brood survival. Nests and broods are collectives of eggs and ducklings, respectively.

* = “Adult survival” submodel calibrated against as narrower range of adult body masses compared to other submodels.

**TABLE A2:** Equation parameters and the points used to generate the equation to convert breeding ground arrival mass (grams) of a female mallard to a probability of creating a first nest.

Original *x*	Translated *x*	*y*	*c*	*b*	*a*
−250	6.84	0.88	−13.4	3.88	–0.260
0	7.08	0.94			
250	7.32	0.97			

*Note:* Original *x*‐ and *y*‐values were extracted from the after‐second‐year female curve of figure 2 of Devries et al. (2008). Units for original *x*‐values were a principal‐components analysis‐based body condition index (BCI), and units for *y*‐values were probabilities. Translated *x*‐values were an approximate conversion of BCI to the natural logarithm of body mass in grams. The equation was fit to (translated *x*, *y*) coordinates. The equation has parameters *c*, *b*, and a with form *y* = *c* + *bx* + *ax* ^2^. All parameters are reported to three significant digits.

**TABLE A3:** Equation parameters and the points used to generate the equation to convert breeding ground arrival mass (grams) of a female mallard to interval between breeding ground arrival and first nest initiation dates.

Original *x*	Original *y*	Translated *x*	Translated *y*	*c*	*b*	*a*
−250	21.5	6.84	26	−1060	338	–26.0
0	15.5	7.08	20			
250	6.5	7.32	11			

*Note:* Original *x*‐values and original *y*‐values were extracted from the after‐second‐year female curve of figure 3 of Devries et al. (2008). Units for original x‐values were a principal‐components analysis‐based body condition index (BCI), and units for original *y*‐values were days after the first initiated at a site, that is, relative nest initiation date. Translated *x*‐values were an approximate conversion of BCI to the natural logarithm of body mass in grams. Translated y‐values were an approximate conversion of relative nest initiation date to the interval between breeding ground arrival and first nest initiation dates. The equation was fit to (translated *x*, translated *y*) coordinates. The equation has parameters *c*, *b*, and *a* with form *y* = *c* + *bx* + *ax* ^2^. All parameters are reported to three significant digits.

## Appendix B Summary of Additional Changes to the Base Reproduction Model

Regarding further integration of Arnold et al. (2012)‐based adult female mallard survival into the reproduction model, we use the “Combined Reproductive Periods” adult survival equation in Appendix 1 of Arnold et al. (2012). The coefficient values of this equation change based upon an adult's reproductive state. This equation reflects the disproportionate adult mortality observed in female mallards during incubation as compared to other reproductive states (Arnold et al. 2012; Gue et al. 2013). This switch to using the Arnold et al. (2012) equation superseded a component of the Johnson et al. (1987) model where nest failures could lead to adult mortality; we instead treated adult mortality as a possible cause of nest failure. Because of this additional source of nest mortality not considered by nest survival probabilities, we empirically calibrated adult survival‐independent daily nest survival probability to be 0.947. This probability, along with the additional nest mortalities from adult mortalities, maintained value close to the mean overall nest survival rate calculated by Johnson et al. (1987), 0.119. Another effect of implementing the Arnold et al. (2012) adult survival equation was a small amount of adult mortality during the brooding phase, contributing to additional brood failures not captured in the original Johnson et al. (1987) model. To account for this additional source of brood failure, we empirically calibrated adult survival‐independent brood survival probability as 0.751. That probability translated to an overall brood survival rate with the default model parameterization of 0.740, the brood survival rate used by Johnson et al. (1987).

We summarize other modifications to the Johnson et al. (1987) model structure and base parameters below. The model description in Appendix A contains further details of how we implemented each modification. We added a component for initiation probability of a first nest and initiation date of the first nest, based on body mass, derived from Devries et al. (2008). Incubation period was converted from a constant 26 days to a random variable sampled from a truncated normal distribution with a mean of 26 days, SD of 2.6 days, minimum of 20 days, and a maximum of 31 days. That SD is a midpoint between the SDs reported by Feldheim (1997) and Wells‐Berlin et al. (2005), and the minimum and maximum are based on Feldheim (1997). We added egg hatchability, a component that is present in the Hoekman et al. (2002) mallard population model, by sampling the hatching success of each from a Bernoulli distribution with a probability of 0.91 (rounding to two decimal places based on cited sources), a probability calculated by Johnson et al. (1992) and used by Hoekman et al. (2002). In the Johnson et al. (1987) model, brood survival has an “entire brood loss” probability that is sampled stochastically using a probability of 0.740. That model also uses a static multiplier of 0.540 for duckling survival for broods that were not completely lost. For brood survival, we increased the survival probability to 0.751 to account for adult mortality during the brooding period. We randomly sampled duckling survival assuming an average proportion of 0.540 survived. The overall target duckling survival, including entire brood loss, was 0.400 (0.740 × 0.540). We implemented a date‐dependent renesting interval based on Arnold et al. (2010); we also used the renesting probability function from Arnold et al. (2010), which depends on adult body condition and initiation date of the failed nest.

## Appendix C Additional Mallard Migration and Energetic Model (MMEM) Details

### Modeling Details

Regarding breeding ground arrival dates and arrival body masses of adult mallards, both of those values in the MMEM are derived using sampling distributions and parameters based on the Johnson et al. (1987) mallard reproduction model, the base model for the present reproduction modeling effort. Arrival dates across 80,000 (that is, 80 replicates of 1,000 birds) unoiled birds from the January MMEM starting states range from 16 March to 10 May, with a mean arrival date of 12 April (West et al. 2026a, 2026b). These parameter values essentially match those used by Johnson et al. (1987) to parameterize a truncated Poisson distribution for arrival dates. A slight difference in arrival date sampling employed by the MMEM is that birds making fewer migratory stopovers arrived, on average, earlier than birds making more stopovers. This variation in arrival dates based on number of stopovers was implemented using Poisson distributions with different λ values for each possible number of stopovers (0–3 stopovers, 4 different λ values; West et al. 2026a). Across 80,000 unoiled birds from the January MMEM starting states, breeding ground arrival body masses have a mean of 1193 g, a standard deviation (SD) of 77 g, and a range of 936–1443 g (West et al. 2026b). The Johnson et al. (1987) reproduction model samples initial body masses for adult female mallards from a normal distribution with a mean of 1200 g and SD of 80 g. The second stage of the MMEM starts by sampling arrival body mass from a truncated normal distribution with a mean of 1200 g, SD of 80 g, minimum of 1000 g, and maximum of 1400 g (West et al. 2026a), but the values in the final data shift slightly during the simulation reruns conducted during the third MMEM stage; see the appendix of West et al. (2026a) for details.

Mallards in the MMEM start the simulation with initial lipid reserves (median = 158 g, range = 10–334 g, West et al. 2026a; West et al. 2026b). They almost always arrive at the breeding grounds with greater lipid reserves than the starting amount if they do not become oiled (98.1% of individuals have increased lipid mass; median arrival lipid reserves for unoiled birds = 251 g, range = 47–441 g; derived from West et al. 2026b). The only source of mortality in this MMEM is starvation, which occurs if a mallard's lipid reserves fall below 10 g (West et al. 2022, 2026a).

Mallard energetics in the MMEM are partially mediated by air temperature via the energetic cost of thermoregulation. Air temperature data used in the model comprise weighted average values across multiple years and do not represent any single calendar year. Besides thermoregulation, other energetic costs are incurred by basal metabolism, flight, and miscellaneous activity. The positive component of energetic balance is termed energetic gain and comprises energy gained from food; this value varies among individuals and changes when an individual migrates to a new location.

Oiled mallards in the MMEM experience up to three energetic effects of oiling: (1) increased cost of thermoregulation, (2) increased cost of flight, and (3) decrease in energetic gain. At the beginning of each MMEM simulation where birds become oiled, the magnitudes of oiling effects for each of the three affected energetic parameters are randomly sampled from truncated normal distributions; trace oiling simulations result in less severe energetic deficits than (West et al. 2026a). Within a given MMEM simulation incorporating oiling, all birds have the same set of 3 randomly sampled alterations to thermoregulation, flight, and energetic gain.

All birds in a simulation either remain unoiled or have the same oiling level during a 14‐day period of active oiling. After that period, energetic effects of active oiling cease (West et al. 2026a). This 14‐day duration is a conservative estimate based on self‐cleaning rates in laboratory studies of lightly oiled birds assessing oil's impacts on energetic costs of thermoregulation (Hartung 1967) and flight (Maggini et al. 2017). The 14‐day migration delay in the second January oiling event matches the duration of oiling.

### Simulation Hierarchy

As a reminder, Stage 1 of the MMEM deals with migration scheduling, Stage 2 deals with energetics and daily body masses in the absence of oil spills. Regarding the specifics of Stage 3 results, they are divided into two components: primary results incorporating stochastic sampling of oiling effects, and the results of a sensitivity analysis (West et al. 2026a, 2026b). We used the primary results of the third stage of the MMEM as inputs for the current reproduction modeling effort.

The base of the Stage 3 (oil spill‐related) MMEM simulations are 2 sets of 80 replicate starting states (80 for January, 80 for March), each comprising 1000 adult female mallards, generated by Stage 2 simulations (160 total Stage 2 simulations; West et al. 2026a). A starting state includes initial body mass, initial lipid mass, and migration schedules; these values differ among replicates due to stochastic processes in Stages 1 and 2 of the MMEM (Appendix A of West et al. (2026a)). The first set of 80 replicate starting states has no explicit restrictions on migration schedules; each of those replicates is used as a set of starting states for the January oiling events, making those 80 replicates the “January MMEM starting states” (Figure 2). The second set of 80 replicates only contains birds that depart the wintering area after the start date of the March oil spill, 6 March, making those 80 replicates the “March MMEM starting states.”

The MMEM simulates the 6 combinations of 2 oiling levels (trace, light) × 3 oiling events (January event with no migration delay, January event with a 14‐day migration delay, March event); it also includes 2 unoiled baselines for each of the January and March spill timings (Figure 2 in main text; West et al. 2026a). Due to the lack of a mechanism for unoiled birds to have delayed migration, the unoiled baseline for January does not incorporate a migration delay. For a given oil spill timing, each oiling level × oiling event or unoiled baseline uses the same 80 MMEM starting states. This structure results in a total of 640 oiling level × oiling event × MMEM starting state combinations (Figure 2 in main text). This use of the same replicates for multiple oiling level × oiling event combinations and the corresponding unoiled baseline within a given oil spill timing means that the results of the MMEM can be treated as repeated measures.

An additional hierarchy layer in the MMEM is the use of two variations of trace and light oiling levels: one where oiled birds experience increased energetic costs for thermoregulation and flight, and second where oiled birds have decreased energetic gain in addition to increased energetic costs for thermoregulation and flight (West et al. 2026a). In the reproduction model presented here, we only included the trace and light oiling variations that incorporate decreased energetic gain, as we assumed that an effect on energetic gain was a more realistic scenario.

## Appendix D Derivation of Production Scenario Parameters

To develop the medium and high production scenarios described in the “Reproduction model scenarios and runs” section, we started with the following equation for population growth rate:

(D1)
$$\lambda =\left(S_{B}+D_{F}\right)S_{N}$$

where *λ* was female‐based population growth rate, *S*_*B*_ was survival rate of adult females during the simulated breeding season, *D*_*F*_ was production of female ducklings per adult female that arrived at the breeding grounds in the unoiled baseline simulations, and *S*_*N*_ was survival rate of all female individuals during the nonbreeding season following the simulated breeding season. Following the mallard population model of Hoekman et al. (2002), we assumed a value of 0.800 for *S*_*N*_. However, we acknowledge that other sources estimate lower (Amundson et al. 2013; Coluccy et al. 2008) or higher (Arnold et al. 2012) values for *S*_*N*_, and that mallard population growth rates are generally sensitive to changes in nonbreeding season survival of female mallards (Amundson et al. 2013; Coluccy et al. 2008; Hoekman et al. 2002). We calculated *D*_*F*_ as:

(D2)
$$D_{F}=P_{F}D_{\mathrm{tot}}=P_{F}\left(N_{D}/N_{B}\right)$$

where *P*_*F*_ was the proportion of surviving ducklings that were female, *D*_*tot*_ was production of all ducklings per adult female, *N*_*D*_ was the number of surviving ducklings produced by a population, and *N*_*B*_ was the population size of breeding females at the start of the breeding season. Following Johnson et al. (1987) and Hoekman et al. (2002), we assumed a value of 0.500 for *P*_*F*_. For our medium and high production scenarios, our goal was to provide parameters for a combined version of Equations D1 and D2 solved for *D*_*tot*_:

(D3)
$$D_{\mathrm{tot}}=\left(\frac{\lambda }{S_{N}}-S_{B}\right)/P_{F},$$

then alter reproduction model parameters to arrive at a determined value for *D*_*F*_. We targeted values for *λ* of 1.00 and 1.05 for the medium and high production scenarios, respectively (rounding to three significant digits/2 decimal points intentional). A *λ* value of 1.00 represented a stable population, and a *λ* value of 1.05 was derived from Midcontinent mallard population estimates for the most recent period of Midcontinent mallard population increase prior to this modeling study, 2005–2016 (USFWS 2025). An increase from an estimated 6.7–11.8 million mallards over the 11‐year span from 2005 to 2016 would require a *λ* value of ~1.05 as 6.7 million × 1.05^11^≈11.8 million; note that this calculation is not exact due to rounding error. We assumed a value of 0.725 for *S*_*B*_ for the medium production scenario, as this is the midpoint between the bounds of adult survival (0.700–0.750) used by Johnson et al. (1987). We assumed a value of 0.757 for *S*_*B*_ for the high production scenario, the survive rate of adult females estimated by Devries et al. (2003), one of the largest, most geographically diverse studies of breeding female mallard survival in the Prairie Pothole Region (Baldassarre 2014). The adult female survival rate that Devries et al. (2003) calculate is also based on data collected during a period of Midcontinent mallard population increase, 1993–1998 (USFWS 2025). Using the above values for *λ*, *S*_*N*_, and *S*_*B*_ in Equation D3 resulted in target values of 1.05 and 1.11 for *D*_*tot*_. We accomplished alterations to *S*_*B*_ and *D*_*F*_ (Equation D2) in the model by altering (1) adult daily survival rate (DSR), (2) nest daily survival rate conditional upon adult survival (“conditional nest DSR”), (3) overall brood survival rate conditional upon adult survival (“conditional brood survival rate”), and (4) overall survival rate of individual ducklings conditional upon at least partial brood survival (“conditional duckling survival rate”). Alterations to all of the above survival rates were made using the equation:

(D4)
$$R_{A}=1-\left(1-R_{0}\right)M_{R}$$

where *R*_*A*_ was an altered survival rate, *R*_*0*_ was a survival rate from the base model (that is, the low production scenario), and *M*_*R*_ was a multiplier applied to 1 − *R*_*0*_ (that is, the mortality rate) to convert *R*_*0*_ to *R*_*A*_. Somewhat counterintuitively, a lower value of *M*_*R*_ resulted in a higher altered survival rate (*R*_*A*_). Within a production scenario, both conditional brood and duckling survival rates shared the same value for *M*_*R*_. Both within and between production scenarios, values of *M*_*R*_ for adult DSRs, conditional nest DSRs, and brood/duckling survival all differed from each other (Table D1). To reach the target values of *S*_*B*_ and *D*_*F*_ for the medium and high production scenarios, we altered values for *M*_*R*_ across the various survival rates. We altered these values such that (1) *S*_*B*_ was within ±0.002 of the target value and (2) nest and duckling survival rates changed by as close to the same amount as possible, with a tolerance of ±0.001. We made the decision to increase nest and duckling survival rates equally because, depending on population vital rates and model parameterization, either nest or duckling survival can be the parameter for which *λ* or recruitment is most sensitive (Amundson et al. 2013; Coluccy et al. 2008; Hoekman et al. 2002; Johnson et al. 1987). Empirically determined *M*_*R*_ values of each combination of life stage and relevant production scenario (medium and high) are contained in Table D1. Note that adult survival, nest survival, and duckling survival were not independent from each other in the model. Nest and brood survival depend on the adult female parent's survival. Additionally, increasing nest survival rates decreased adult survival rates by increasing the amount of time adults spent incubating, the reproductive state with the lowest adult survival rates (Arnold et al. 2012; Gue et al. 2013). A consequence of this lack of independence was that a model outcome like nest survival rate could differ if we changed *M*_*R*_ for adult DSRs, regardless of whether we altered *M*_*R*_ for conditional nest DSRs. A consequence of this lack of independence was that *M*_*R*_ for conditional nest DSR was lower for the medium production scenario compared to the high production scenario (Table D1), even though the opposite pattern would be expected given that a lower value for *M*_*R*_ leads to a higher conditional nest DSR.

**TABLE D1:** Mortality rate multipliers (*M*_*R*_) for medium and high production scenarios within a mallard (*Anas platyrhynchos*) reproduction model.

Survival rate	Production scenario	*M*_*R*_
Adult DSR	Medium	0.914
	High	0.795
Conditional nest DSR	Medium	0.524
	High	0.528
Conditional brood and duckling survival rates	Medium	0.647
	High	0.637

*Note:* Values for *M*_*R*_ were applied within the following equation: *R*_*A*_ = 1 – (1 – *R*_*0*_)*M*_*R*_, where *R*_*A*_ was an altered survival rate, *R*_*0*_ was a baseline survival rate, and 1 − *R*_*0*_ was a baseline mortality rate. Survival rate were (1) adult daily survival rate (DSR), (2) nest daily survival rate conditional upon adult survival (“conditional nest DSR”), (3) overall brood survival rate conditional upon adult survival (“conditional brood survival rate”), and (4) overall survival rate of individual ducklings conditional upon at least partial brood survival (“conditional duckling survival rate”). The same value for *M*_*R*_ was used for both conditional brood and duckling survival rates, hence a single table row per production scenario. The medium production scenario corresponded to an estimated population growth rate of 1.00; high corresponded to an estimated population growth rate of 1.05.

## Appendix E Data Summary for Unoiled Baseline Model Results

### Methods

In addition to the six output variables mentioned in Section 2.3. Reproduction model scenarios and runs within the main body of the text, we summarized six model outputs just for the unoiled baseline, specifically rates for (1) nest survival, (2) duckling survival, (3) per‐adult hatching success, (4) per‐adult fledging success, (5) nest initiation, and (6) renesting. Due to the reproduction model structure, any changes to nest survival rate and duckling survival rate under the trace or light oiling levels would be side‐effects of changes in adult survival rates and would be largely captured by the variables total duckling production, per‐adult hatching success rate, and adult survival rate. However, nest survival and duckling survival are key components of mallard reproduction and population models (for example, Amundson et al. 2013; Hoekman et al. 2002; Johnson et al. 1987), so we report them for the base reproduction model. Per‐adult hatching and fledging success rate were production indices that could be used as alternatives to per‐adult duckling production rate. Per‐adult hatching success rate was the proportion of adults that successfully hatched a nest (“hen success” *sensu* Cowardin and Johnson 1979). Per‐adult fledging success rate was the proportion of adults that successfully fledged at least one duckling. Both of those metrics were indices of per‐adult duckling production rate, so we report only per‐adult duckling production rate differences for trace and light oiling levels because it is directly used in the estimation of population growth rate for the unoiled baseline condition (see Equation D1 in Appendix D). For the trace and light oiling level simulations, we considered nests per adult to be a combined indicator of nest initiation and renesting rates. The numerator for nest initiation rate was the number of adults that created at least one nest, and the denominator included only adults that created a nest and/or survived for 26 days after arriving on the breeding grounds. The maximum duration of the prenesting state for unoiled mallards that created a nest was 26 days (see Submodel “Prenesting Interval” in Appendix A). The numerator for renesting rate was the number of adults that created at least two nests, and the denominator included only adults that had their first nest fail, survived that nest failure, and survived either 11 days or until they created a second nest; 11 days was the maximum duration of the postnesting state for mallards that renested (see Submodel “Failed Nest Response” in Appendix A).

We used a two‐step process to generate summary statistics for the summarization of the unoiled baseline. First, we calculated the mean value across all 200 reproduction model replicates for each of the 480 unoiled reproduction model starting states. Next, we calculated the median and [0.025, 0.975] interquantile range (95% IQR) for those means across 80 MMEM replicates for each of the six combinations of production scenario (3) × oil spill timing (2). We only reported these summary statistics for the January unoiled baseline, as median values for birds in the March unoiled baseline differed by a magnitude of ≤ 0.313% from each corresponding January unoiled baseline median. See West et al. (2025) for full raw model results.

### Results

As expected, the medium and high production scenarios achieved their target values for estimated population growth rate, adult survival, and per‐adult duckling production, whereas the low production scenario reflected baseline model parameterization without target benchmarks (Table E1). Nest survival, duckling survival, and overall duckling production increased progressively from the low to the high production scenario, consistent with the adjustments made to breeding‐season survival parameters. In contrast, several components of nesting intensity, e.g., nests per adult, nest initiation rate, and renesting rate, showed overlapping uncertainty intervals across production scenarios, indicating no evidence of differences among them. Mean clutch size similarly exhibited minimal variation across scenarios. Collectively, these results demonstrate that production scenario parameterization produced the expected differences for survival‐ and output‐based metrics, while measures tied to nesting propensity and clutch formation remained comparatively stable across categories.

**TABLE E1:** Summary statistics for a simulation‐based mallard (*Anas platyrhynchos*) reproduction model prior to the incorporation of carry‐over effects.

Variable	Production scenario			Description
	Low	Medium	High	
**Population growth**				
Estimated population growth rate	0.93 [0.93, 0.94]	1.00 [1.00, 1.01]	1.05 [1.04, 1.05]	Estimated single‐year population growth rate*
**Survival rates**				
Adult female survival rate (breeding season only)	0.706 [0.704, 0.708]	0.726 [0.724, 0.728]	0.758 [0.756, 0.760]	Prop. adult females that survived the breeding season
Nest survival rate	0.121 [0.120, 0.122]	0.137 [0.136, 0.138]	0.140 [0.139, 0.141]	Prop. nests that hatched
Duckling survival rate	0.404 [0.402, 0.407]	0.419 [0.417, 0.421]	0.422 [0.420, 0.424]	Prop. ducklings that survived until fledging
**Raw production**				
Total duckling production	914 [900, 926]	1053 [1040, 1066]	1102 [1092, 1116]	Number of fledged ducklings
**Production indices**				
Per‐adult hatching success rate	0.276 [0.273, 0.279]	0.306 [0.303, 0.309]	0.319 [0.315, 0.323]	Prop. adult females that hatched a nest
Per‐adult fledging success rate	0.204 [0.202, 0.207]	0.229 [0.226, 0.231]	0.239 [0.237, 0.242]	Prop. adult females that fledged at least one duckling
Per‐adult duckling production rate	0.91 [0.90, 0.93]	1.05 [1.04, 1.07]	1.10 [1.09, 1.12]	Fledged ducklings per adult female
**Nesting intensity**				
Nests per adult	2.29 [2.26, 2.31]	2.24 [2.22, 2.26]	2.28 [2.26, 2.30]	Number of nests created per breeding female
Nest initiation rate	0.941 [0.939, 0.942]	0.941 [0.939, 0.942]	0.941 [0.939, 0.942]	Prop. adult females that made at least one nest
Renesting rate	0.958 [0.955, 0.961]	0.956 [0.954, 0.960]	0.956 [0.953, 0.959]	Prop. adult females that survived a nest failure and made a second nest
**Clutch size**				
Mean clutch size	9.16 [9.14, 9.19]	9.18 [9.16, 9.21]	9.16 [9.14, 9.19]	Mean clutch size across nests surviving the laying period

*Note:* Each simulation started with 1000 adult female mallards, there were 80 non‐identical replicate starting conditions, and each of those starting conditions was simulated 200 times. Values were summarized in a hierarchical manner. First, we calculated mean across groups 200 simulations with identical starting conditions. Next, we used those means to calculate and report median and [0.025, 0.975] quantiles across the 80 non‐identical replicate starting conditions. Subheadings within the Variable column group similar output variables. “Adult female” refers to after second year individuals; yearling females were not included in the model. Prop. = “proportion of.” * = Estimated single‐year population growth rate for the modeled breeding season and the prior non‐breeding season, assuming a non‐breeding survival rate of 0.800 for all individuals (based on Hoekman et al. 2002).
